# Supplementary material for: Distinguishing gene flow between malaria parasite populations
Source: PLoS Genet. 2021 Dec 20;17(12):e1009335. doi: 10.1371/journal.pgen.1009335 (PMC8726502; doi:10.1371/journal.pgen.1009335)
Supplement: S1 Appendix — Fig A. Positions for filtered SNPs from the Pf3k dataset. (A) SNPs with estimated minor allele frequency > 0.35. (B) SNPs with estimated minor allele frequency > 0.05. Fig B. Number of monoclonal P. falciparum sequences from the Pf3k database included in this study by location. Data includes only those sequences collected between 2009 and 2011 and excludes KEL1 mutants (as described in Methods). Contains information from OpenStreetMap and OpenStreetMap Foundation, which is made available under the Open Database License. Fig C. Coalescent models used to generate simulated sequence data. (A): Constant population size with change from ancestral migration rates to current migration rates at time = g generations in the past. The ancestral migration rate is equal for all location-location pairs and specified as ρij = M × max(ρCDR), where M is a multiplier. Recent migration rates are specified using CDR-estimated mobility data (ρCDR, as described in the Methods and Supplementary Methods) and a multiplier value m. (B): Constant migration rates with exponential decrease in population size from an initial size (Ni) to current size (Nf) starting at time = g generations in the past. (C): Constant population size and constant migration rates. Fig D. CDR-estimated migration rates. (A) Relative migration rates (ρCDR/max(ρCDR)) between 930 districts in Thailand with adequate CDR data. (B) 10 randomly sampled sets of n = 5 neighboring districts with distances between district centroids of approximately 100 km or less. (C) 10 randomly sampled sets of n = 5 distantly-separated districts, selected to include districts > 600 km from randomly chosen a center district. Sampling procedures for the district sets in (B) and (C) and their use for specifying migration rates in the coalescent simulation are described in the Methods and Supplementary Methods. Fig E. Example sets of nearby districts. Figure shows four of ten total randomly selected district sets used to specify migration rates in [file pgen.1009335.s001.pdf]

Supplementary material for Brown et al "Distinguishing gene flow between malaria parasite populations"

Supplementary Methods: Mobile phone-associated human mobility data and estimated parasite migration rates

We analyzed call detail records (CDR) from approximately 11 million mobile phone subscribers in Thailand collected between 1 August 2017 and 19 October 2017. This dataset has contributed to prior studies of dengue transmission [1] and SARS-CoV-2 epidemic dynamics [2]. The researchers had no access to raw CDR data and instead received pre-processed trip count data aggregated over administrative districts. This data ascertains trips by determining each user's most frequently visited mobile network tower for each of two consecutive 24-hour periods. The most-visited tower during the first 24-hour period and the most visited tower during the following 24-hour period are assumed to represent the origin and destination, respectively, of a single trip between the areas serviced by the tower at each node. Trips between towers were aggregated over 930 administrative districts in Thailand. We let $T_{i,j}^{\text{raw}}$ denote the raw, unweighted trips originating from district i and terminating at district j, averaged over all daily observations in the data collection period. We normalize the raw trip counts by calculating the proportion of all outgoing trips from district i that terminate in district j and multiplying this proportion by the estimated total population of the district i.

$$T_{i,j}^{\text{weighted}} = N_i \times \frac{T_{i,j}^{\text{raw}}}{\sum^k T_{i,k}^{\text{raw}}} \tag{1}$$

where N_i is the 2017 population of district i calculated using estimated population density maps from WorldPop [3] and the denominator is the sum of all trips from district i to all districts $k \in \{1, 2, ..., 930\}$. We use T^{weighted} values to approximate the proportion of the parasite population in j that are migrants from i in each generation as

$$\rho_{j,i} = \frac{T_{i,j}^{\text{weighted}} \times \beta_i}{\sum^k T_{k,j}^{\text{weighted}} \times \beta_j} \tag{2}$$

where the denominator is the sum of all trips from districts $k \in \{1, 2, ..., 930\}$ terminating in district j times β_j, the overall prevalence of infection in j. The numerator is the weighted number of daily trips from i to j times β_i, the overall prevalence of infection in i. If we assume that prevalence of infection is equal across locations ($\beta_i = \beta_j$), and that infection does not modify host movement, then

$$\rho_{j,i} = \frac{T_{i,j}^{\text{weighted}}}{\sum^k T_{k,j}^{\text{weighted}}} \tag{3}$$

$\rho_{j,i}$ approximates the average daily proportion of all individuals in district j who were in district i during the preceding 24-hour period. *ms* coalescent simulation specifies migration rates as the proportion of subpopulation j that is made up of migrants from subpopulation i *each generation*. The aggregated CDR mobility data used here is memory-less (i.e. it does not track users across more than two days) and thus cannot directly estimate the total number of individuals making trips from i to j over multiple days or weeks. Generation time in *P. falciparum* is difficult to measure, but often assumed to be 2-6 generations per year [4, 5]. We use the multiplier m to account for these two considerations and denote the resulting CDR-estimated migration rate as

$$\rho_{j,i}^{CDR} = m \times \rho_{j,i} \tag{4}$$

If the generation time is assumed to be 120 days, and if individuals making trips on each day are completely unique (i.e. the trips on each of the 60 days are made by 60 non-overlapping groups of individuals),

m would equal 120. We ran ms simulations with with values of $m \in 5, 15, 50$ to account for uncertainty in both generation time and extent to which daily aggregated mobility may over- or under-estimate the total number of individuals contributing to migration over the course of a each generation. The distribution of migration estimated rates between 930 districts in Thailand is shown in Figure D.

To approximate migration over different spatial scales, we sampled sets of 5 districts that were either nearby one another ("local" migration) or distantly separated ("subnational" migration). To sample nearby districts, we randomly selected a single district and then randomly selected four other districts with centroids less than 100 km from the centroid of the first district (Figures D and E). To sample distantly separated districts, we selected a single district and then selected four other districts at least 600 km away from the first district (Figures D and F). We sampled 10 local and 10 subnational five-district sets and used these to generate migration matrices with entries $m \times \rho_{j,i}$, as described above.

Supplementary Figures

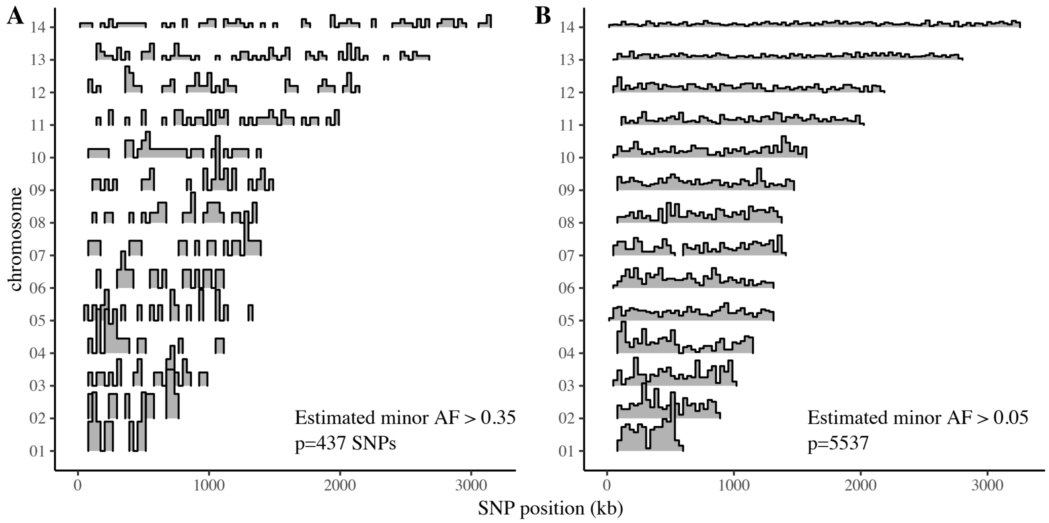

Fig A: Positions for filtered SNPs from the Pf3k dataset. (A) SNPs with estimated minor allele frequency > 0.35. (B) SNPs with estimated minor allele frequency > 0.05.

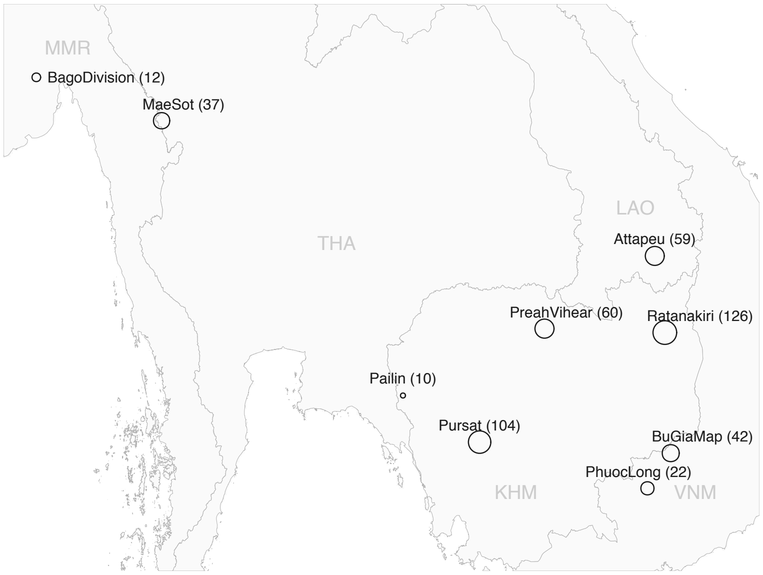

Fig B: Number of monoclonal *P. falciparum* sequences from the Pf3k database [6] included in this study by location. Data includes only those sequences collected between 2009 and 2011 and excludes KEL1 mutants (as described in Methods). Contains information from OpenStreetMap and OpenStreetMap Foundation, which is made available under the Open Database License.

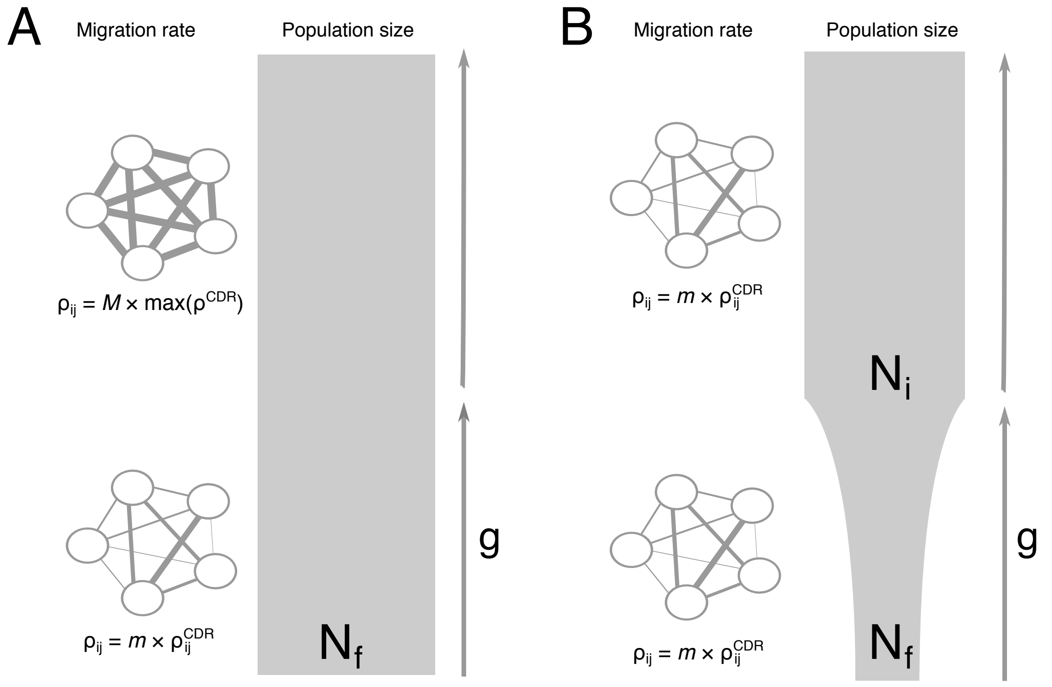

Fig C: Coalescent models used to generate simulated sequence data. (A): Constant population size with change from ancestral migration rates to current migration rates at time = g generations in the past. The ancestral migration rate is equal for all location-location pairs and specified as $\rho_{ij} = M \times \max(\rho^{CDR})$, where M is a multiplier. Recent migration rates are specified using CDR-estimated mobility data (ρ^{CDR}, as described in the Methods and Supplementary Methods) and a multiplier value m. (B): Constant migration rates with exponential decrease in population size from an initial size (N_i) to current size (N_f) starting at time = g generations in the past. (C): Constant population size and constant migration rates

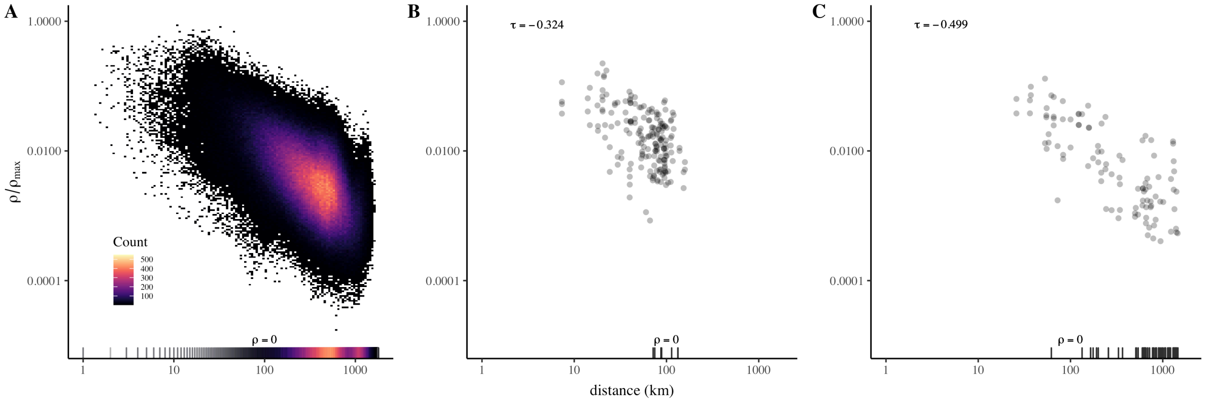

Fig D: CDR-estimated migration rates. (A) Relative migration rates ($\rho_{CDR}/\max(\rho_{CDR})$) between 930 districts in Thailand with adequate CDR data. (B) 10 randomly sampled sets of n=5 neighboring districts with distances between district centroids of approximately 100 km or less. (C) 10 randomly sampled sets of n=5 distantly-separated districts, selected to include districts > 600 km from randomly chosen a center district. Sampling procedures for the district sets in (B) and (C) and their use for specifying migration rates in the coalescent simulation are described in the Methods and Supplementary Methods.

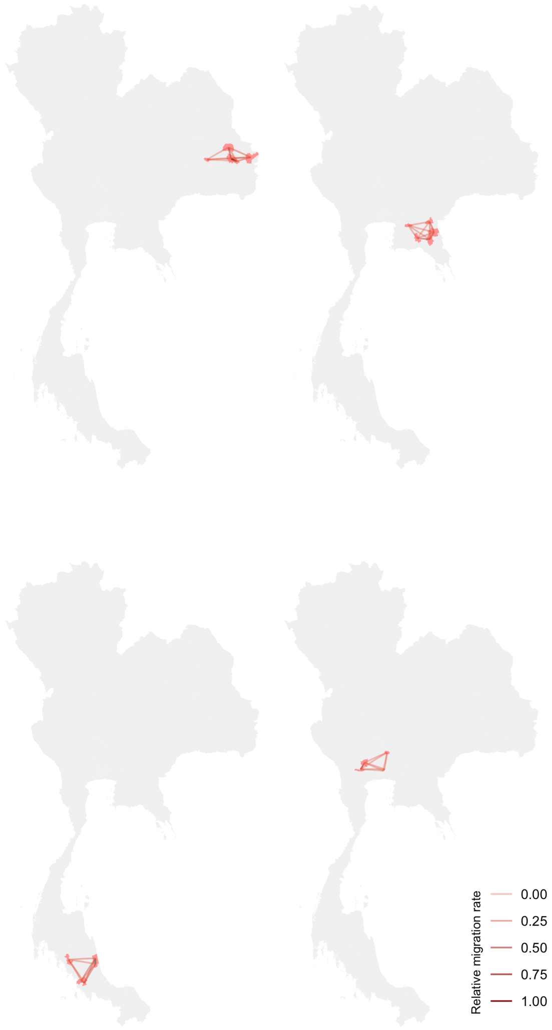

Fig E: Example sets of nearby districts. Figure shows four of ten total randomly selected district sets used to specify migration rates in the coalescent model (corresponding to Panel B in Figure D). Contains information from OpenStreetMap and OpenStreetMap Foundation, which is made available under the Open Database License.

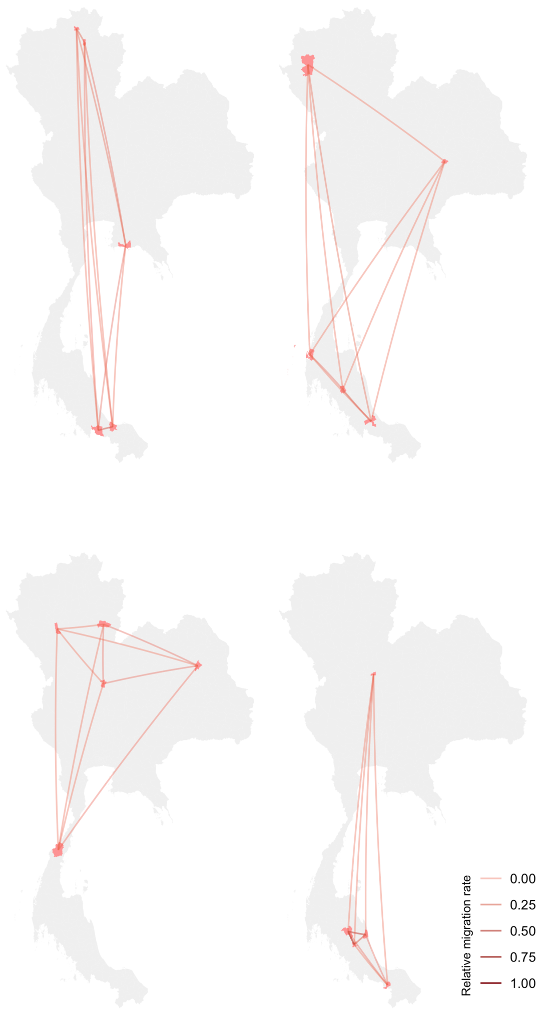

Fig F: Example sets of distant districts. Panels show four of ten total randomly selected district sets used to specify migration rates in the coalescent model (corresponding to Panel C in Fig D) Contains information from OpenStreetMap and OpenStreetMap Foundation, which is made available under the Open Database License.

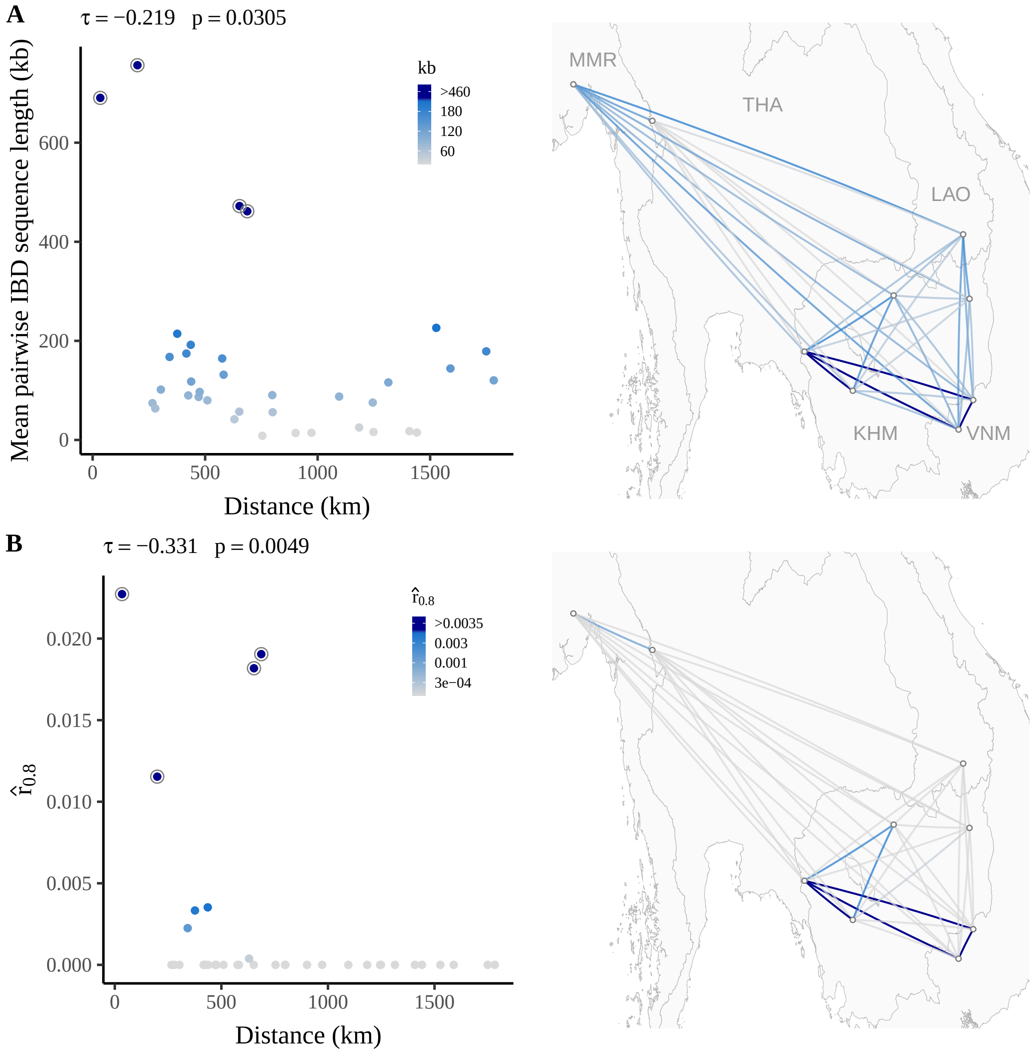

Fig G: Distance versus alternative population-level estimates of relatedness. (A) Distance versus mean pairwise IBD sequence length when only the longest IBD tract lengths are considered ($> 95^{th}$ percentile, equal to tract length $\geq$ 285.64 kb). (B) Distance versus $\widehat{r}_{0.8}$, the proportion between-location individual-individual pairs with $r > 0.8$. Contains information from OpenStreetMap and OpenStreetMap Foundation, which is made available under the Open Database License.

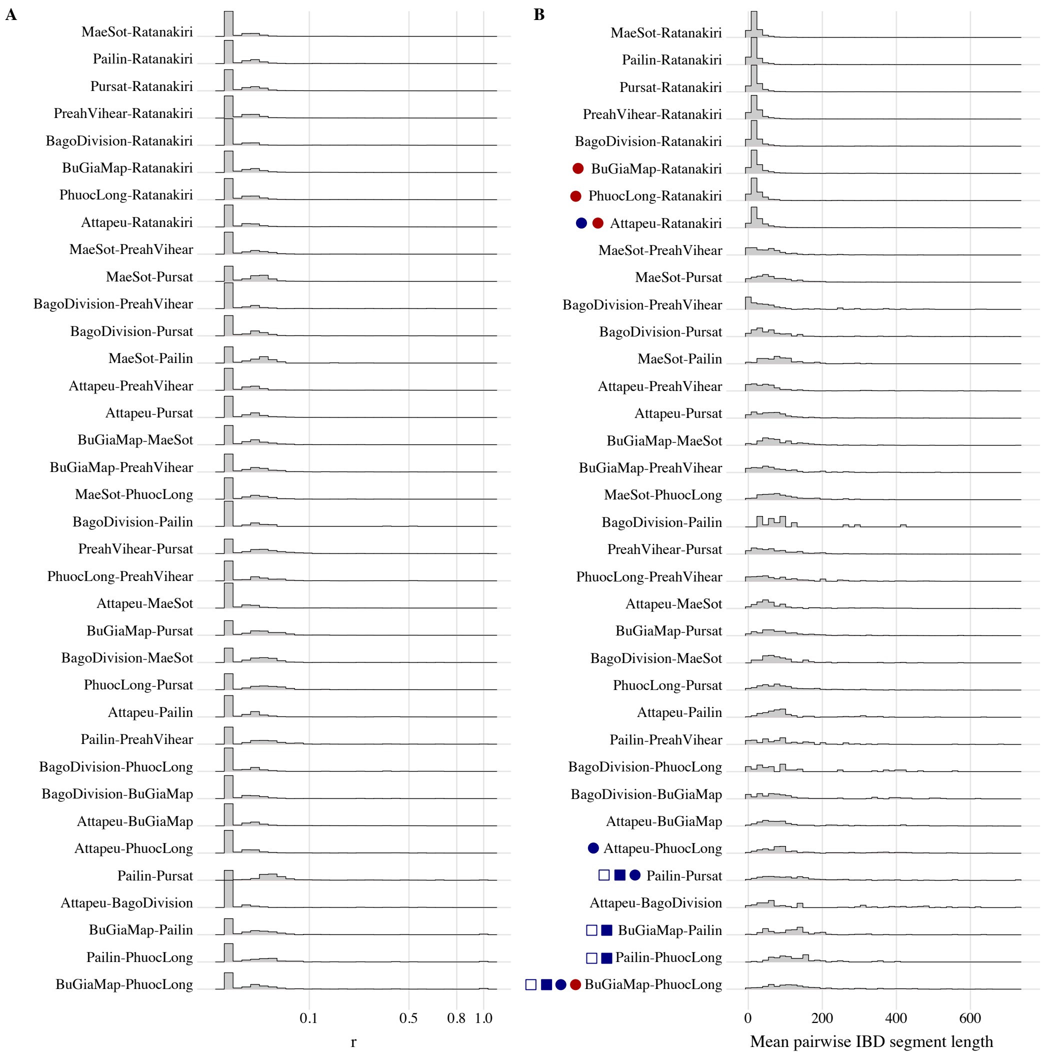

Fig H: Between-location relatedness for *P. falciparum* individual-individual pairs. (A) Distribution of r values (proportion of SNPs that are IBD between pairs) for between-location individual-individual pairs across 36 location-location pairs in the Great Mekong Subregion. (B) Distribution of mean pairwise IBD segment length (the mean length of shared IBD tracts for individual-individual pairs) for the same location-location pairs. Symbols show location-location pairs with the highest estimated gene flow per F_{ST} (red circles), $\widehat{r}_{0.1}$ (blue circles), $\widehat{r}_{0.5}$ (open blue squares), and mean pairwise IBD sequence length (as described in Fig G, filled blue squares).

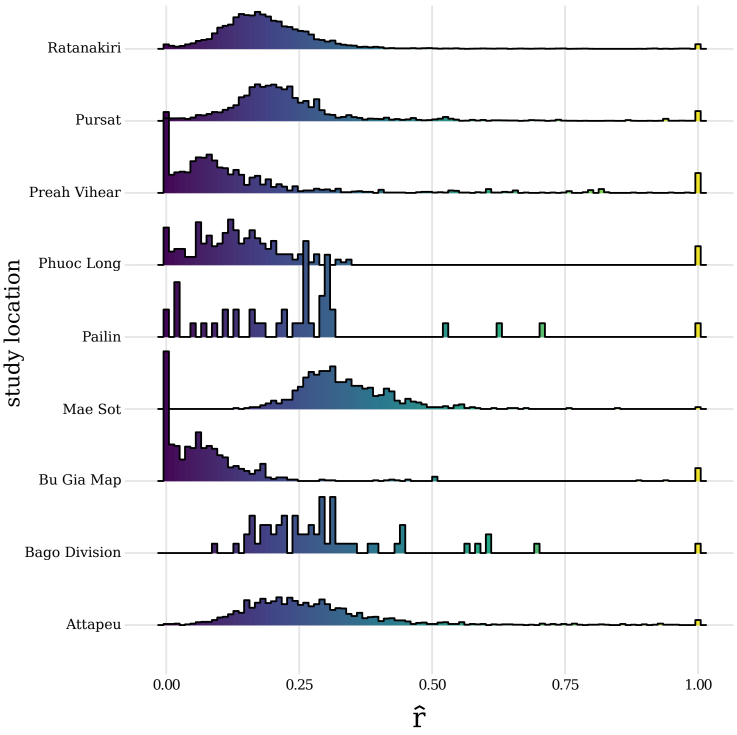

Fig I: Pairwise relatedness for intra-location individual-individual pairs. Plots show distribution of intra-location r values for 9 locations (proportion of SNPs that are IBD between within-location pairs of sequences) included from the Pf3k dataset.

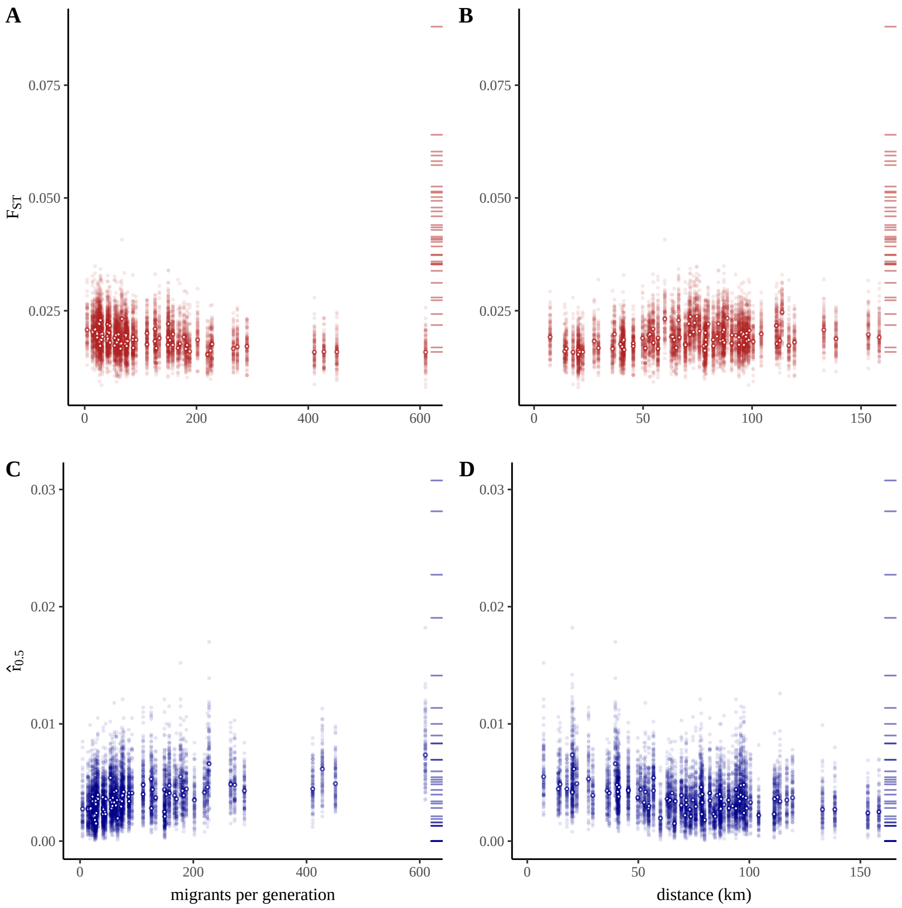

Fig J: Observed values for F_{ST} and $\widehat{r}_{0.5}$ coalescent-simulated sequence data (local migration, model A). Data was simulated using constant population size and instantaneous change from ancestral migration rates to recent migrations at $time = g$ generations in the past (as described in Panel A of Fig C). Model parameters: recombination rate, $\phi = 0.7$, multiplier for recent migration rates, $m = 15$; multiplier for ancestral migration rates, $M = 5$; population size, $N_f = 500$; time since ancestral migration rates, $g = 10$ generations. (A) and (B): Observed F_{ST} values compared to specified migration rates and distance between districts, respectively. (C) and (D): Observed $\widehat{r}_{0.5}$ values compared to specified migration rates and distance between districts, respectively. Filled circles show values for 1000 independent simulations using 10 randomly sampled district sets specifying CDR-estimated migration rates (as shown in Figure D). Open circles show the mean F_{ST} or $\widehat{r}_{0.5}$ values for each migration rate or distance. Rug plot on y-axis shows estimated F_{ST} or $\widehat{r}_{0.5}$ values obtained from the Pf3k *P. falciparum* data (for comparison with the model-derived values).

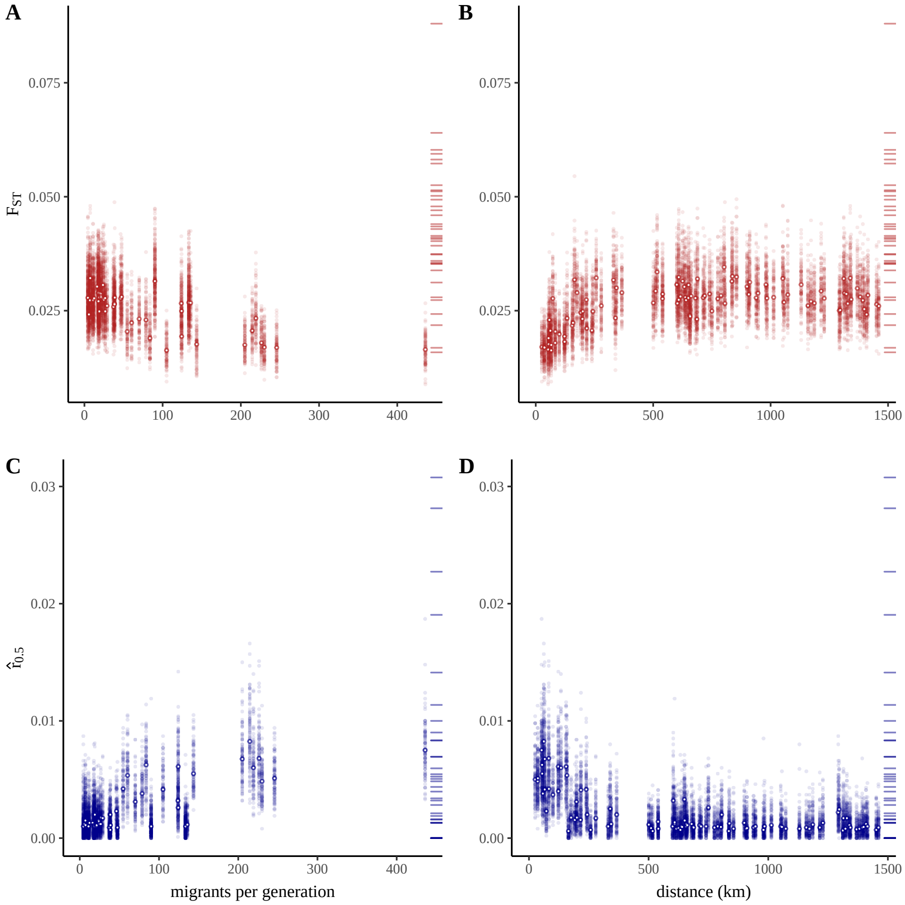

Fig K: Observed values for F_{ST} and $\widehat{r}_{0.5}$ coalescent-simulated sequence data (subnational migration, model A). Data was simulated using constant population size and instantaneous change from ancestral migration rates to recent migrations at $time = g$ generations in the past (as described in Panel A of Fig C). Model parameters: recombination rate, $\phi = 0.7$, multiplier for recent migration rates, $m = 15$; multiplier for ancestral migration rates, $M = 5$; population size, $N_f = 500$; time since ancestral migration rates, $g = 10$ generations. (A) and (B): Observed F_{ST} values compared to specified migration rates and distance between districts, respectively. (C) and (D): Observed $\widehat{r}_{0.5}$ values compared to specified migration rates and distance between districts, respectively. Filled circles show values for 1000 independent simulations using 10 randomly sampled district sets specifying CDR-estimated migration rates (as shown in Fig D). Open circles show the mean F_{ST} or $\widehat{r}_{0.5}$ values for each migration rate or distance. Rug plot on y-axis shows estimated F_{ST} or $\widehat{r}_{0.5}$ values obtained from the Pf3k *P. falciparum* data (for comparison with the model-derived values).

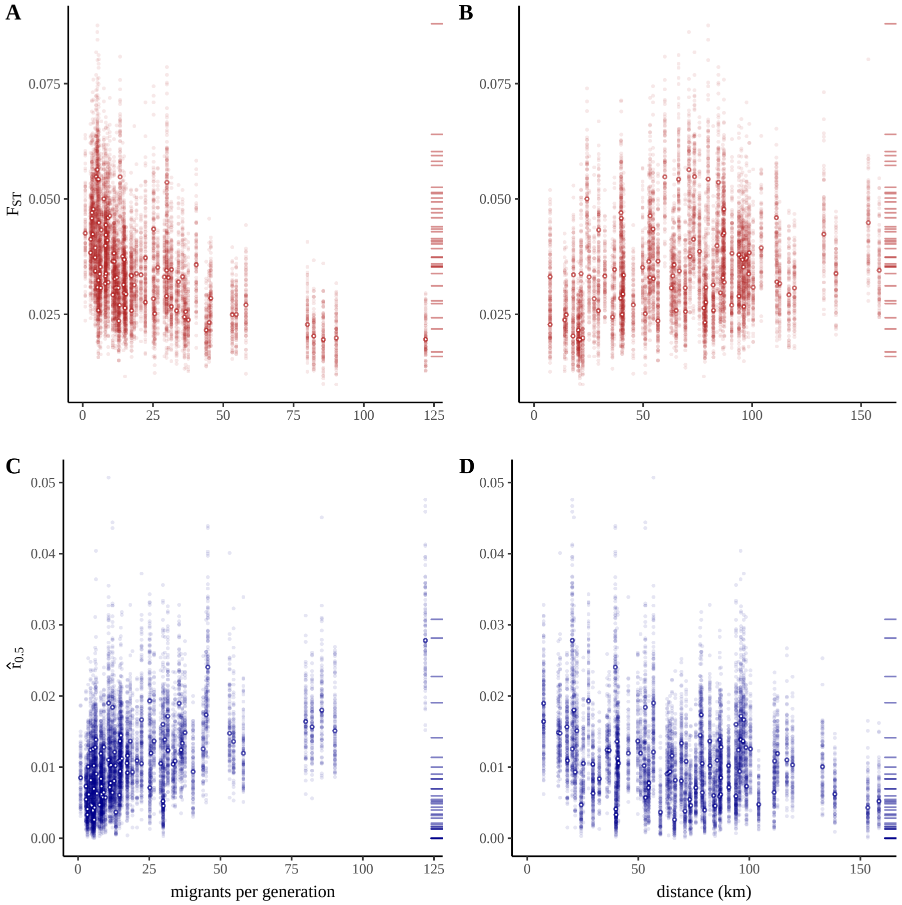

Fig L: Observed values for F_{ST} and $\widehat{r}_{0.5}$ coalescent-simulated sequence data (local migration, model B). Data was simulated using a coalescent model with a constant set of migration rates and exponential decrease from an ancestral population size (N_i) to current populations size (N_f) starting $time = g$ generations in the past (as described in Panel B of Fig C). Migration rates are specified using CDR-estimated mobility between nearby districts in Thailand separated by approximately 100 km or less ("local" migration). Model parameters: recombination rate, $\phi = 0.7$, multiplier for migration rates, $m = 15$; final population size, $N_f = 100$; ancestral population size, $N_i = 1000$; time since onset of exponential decrease in population size, $g = 50$ generations. (A) and (B): Observed F_{ST} values compared to specified migration rates and distance between districts, respectively. (C) and (D): Observed $\widehat{r}_{0.5}$ values compared to specified migration rates and distance between districts, respectively. Filled circles show values for 1000 independent simulations using 10 randomly sampled district sets specifying CDR-estimated migration rates (as shown in Fig D). Open circles show the mean F_{ST} or $\widehat{r}_{0.5}$ values for each migration rate or distance. Rug plot on y-axis shows estimated F_{ST} or $\widehat{r}_{0.5}$ values obtained from the Pf3k *P. falciparum* data (for comparison with the model-derived values).

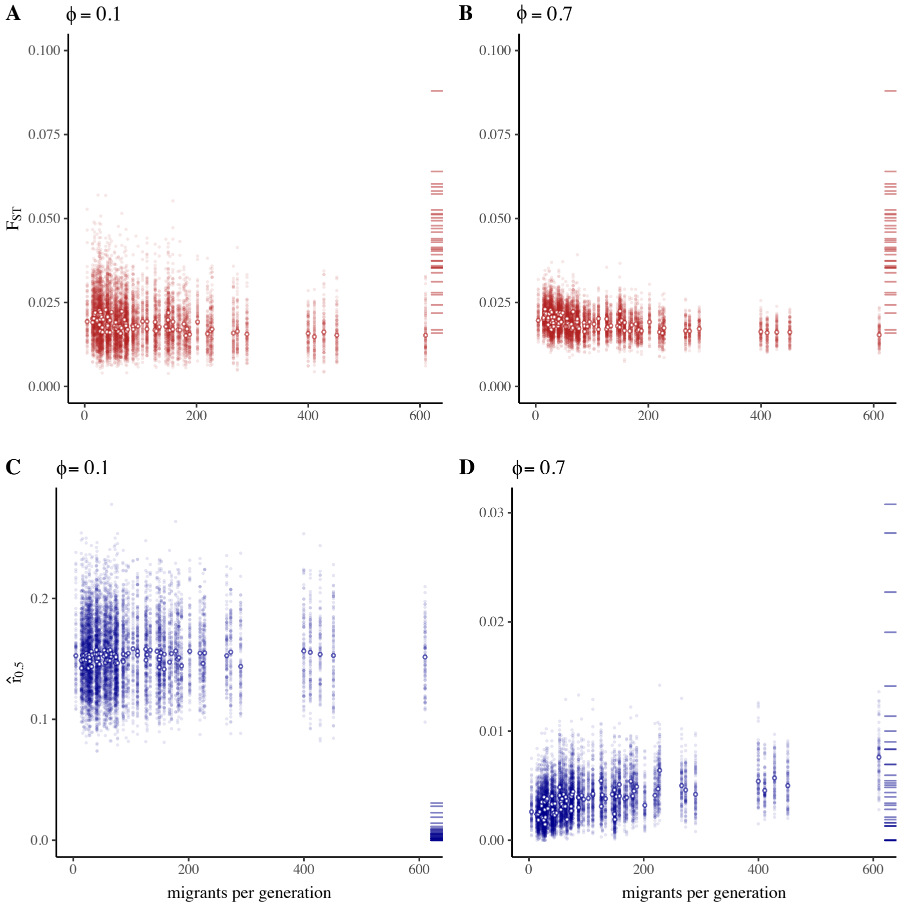

Fig M: Sensitivity analysis for recombination rate ϕ used in coalescent simulations (local migration, model A). Model parameters: recombination rate, ϕ =0.1 or 0.7, multiplier for recent migration rates $m = 15$, multiplier for ancestral migration rates $M = 5$; final population size, $N_f = 500$; time since ancestral migration rates, $g = 10$ generations. (A) and (B): Observed F_{ST} values compared to specified migration rates between districts for ϕ =0.1 and 0.7, respectively. (C) and (D): Observed $\widehat{r}_{0.5}$ values compared to specified migration rates. Filled circles show values for 1000 independent simulations using 10 randomly sampled district sets specifying CDR-estimated migration rates. Open circles show the mean F_{ST} or $\widehat{r}_{0.5}$ values for each migration rate or distance. Rug plot on y-axis shows estimated F_{ST} or $\widehat{r}_{0.5}$ values obtained from the Pf3k *P. falciparum* data (for comparison with the model-derived values).

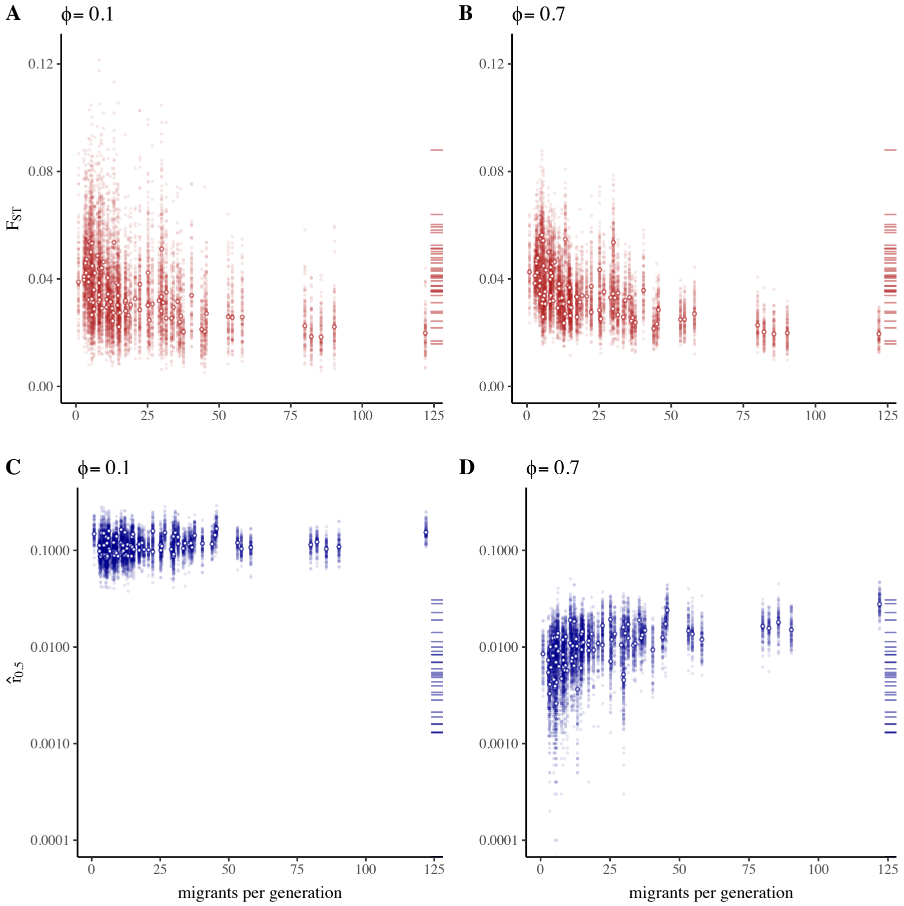

Fig N: Sensitivity analysis for recombination rate ϕ used in coalescent simulations (local migration, model B). Model parameters: recombination rate, ϕ =0.1 or 0.7, multiplier for migration rates $m = 15$; final population size, $N_f = 100$; ancestral population size, $N_i = 1000$; time since onset of exponential decrease in population size, $g = 50$ generations. (A) and (B): Observed F_{ST} values compared to specified migration rates between districts for ϕ =0.1 and 0.7, respectively. (C) and (D): Observed $\widehat{r}_{0.5}$ values compared to specified migration rates. Filled circles show values for 1000 independent simulations using 10 randomly sampled district sets specifying CDR-estimated migration rates. Open circles show the mean F_{ST} or $\widehat{r}_{0.5}$ values for each migration rate or distance. Rug plot on y-axis shows estimated F_{ST} or $\widehat{r}_{0.5}$ values obtained from the Pf3k *P. falciparum* data (for comparison with the model-derived values).

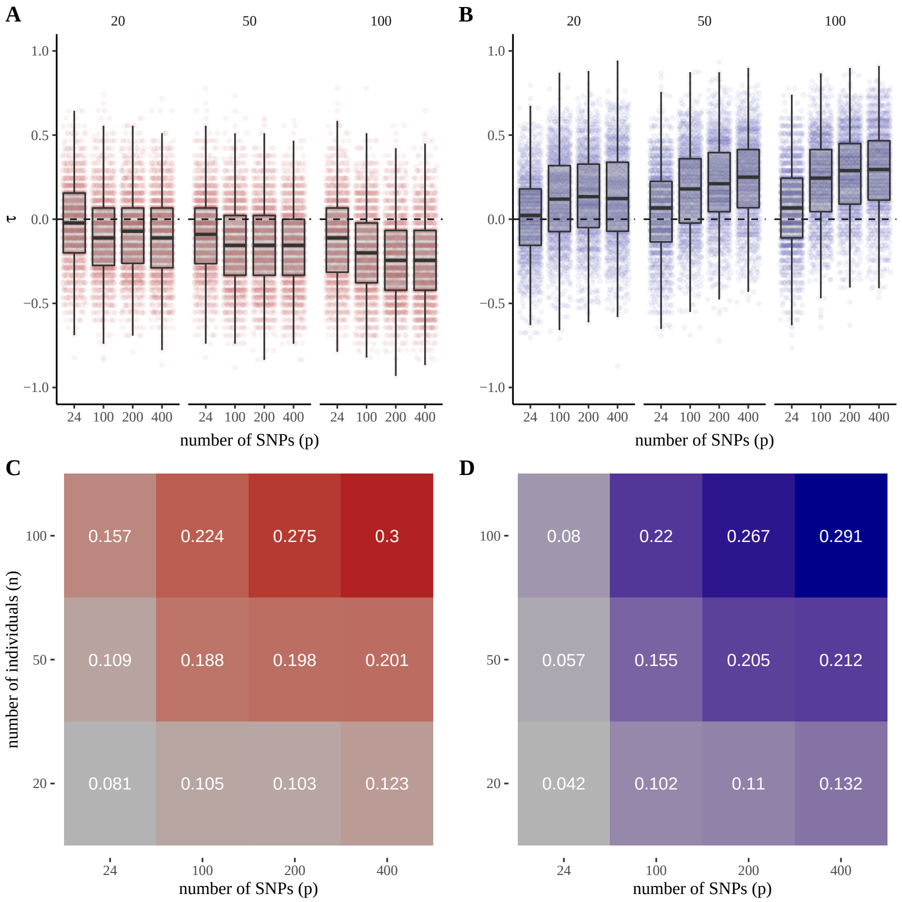

Fig O: Gene flow versus migration rate for coalescent-simulated sequence data (local migration, model A). Migration rates are estimated using CDR data for neighboring districts in Thailand (separated by ≤ 100 km). (A) and (B) correlation between migration rate (as specified in the coalescent model) and observed values of F_{ST} and $\widehat{r}_{0.5}$, respectively. Points show τ values for 1000 independent simulation replicates. (C) and (D): proportion of simulation replicates where Mantel-estimated p-values for observed τ values are ≥ 0.05, by number of SNPs (p) and number of individuals (n).

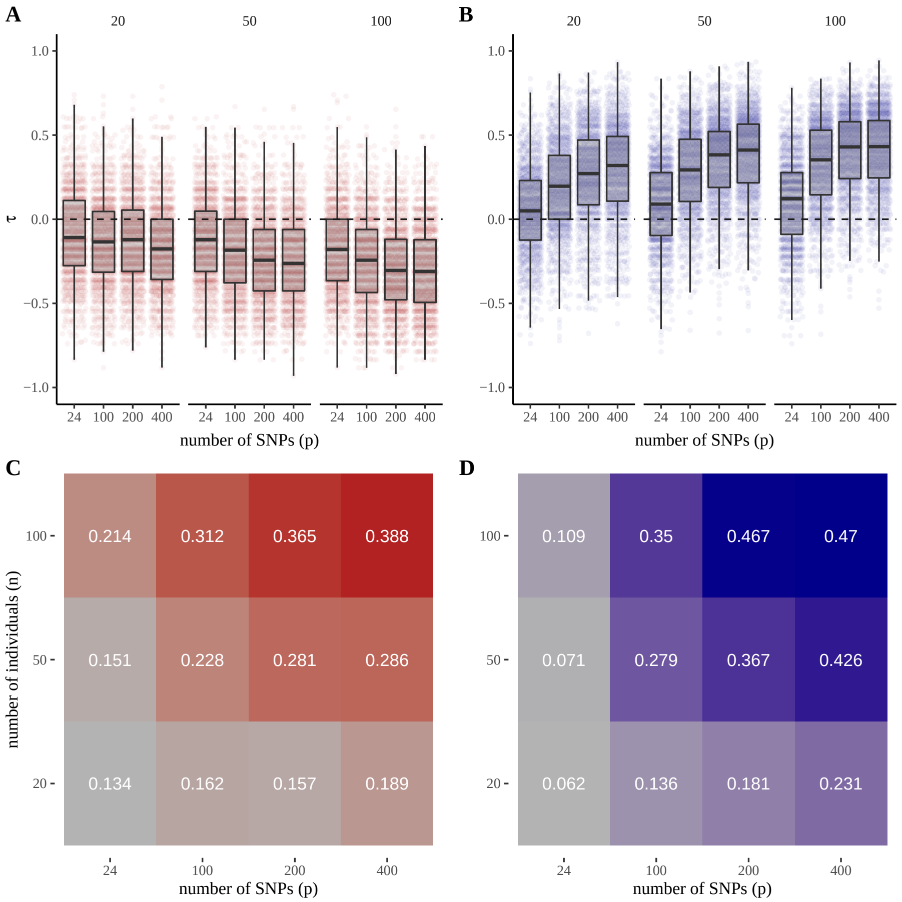

Fig P: Gene flow versus migration rate for coalescent-simulated sequence data (subnational migration, model A). Migration rates are estimated using CDR data for distant districts in Thailand. (A) and (B) correlation between migration rate (as specified in the coalescent model) and observed values of F_{ST} and $\widehat{r}_{0.5}$, respectively. Points show τ values for 1000 independent simulation replicates. (C) and (D): proportion of simulation replicates where Mantel-estimated p-values for observed τ values are ≥ 0.05, by number of SNPs (p) and number of individuals (n).

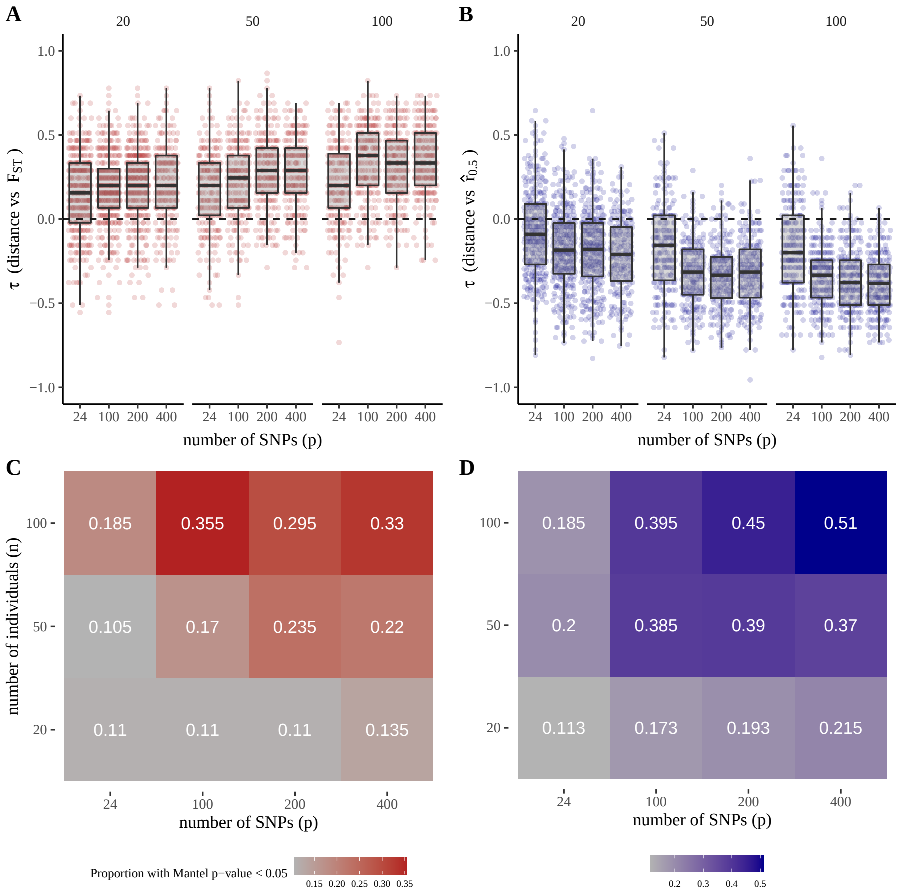

Fig Q: Gene flow versus distance for simulated sequence data (local migration, model B). Data was simulated using a coalescent model with a constant set of migration rates and exponential decrease from an ancestral population size (N_i) to current populations size (N_f) starting $time = g$ generations in the past (as described in Panel B of Figure C). Migration rates are estimated using CDR data for neighboring districts in Thailand (separated by $\geq$ 100 km). (A) and (B): correlation between distance and observed values of F_{ST} and $\widehat{r}_{0.5}$, respectively. Points show τ values for 1000 independent simulation replicates. (C) and (D): proportion of simulation replicates where Mantel-estimated p-values for observed τ values are ≥ 0.05, by number of SNPs (p) and number of individuals (n).

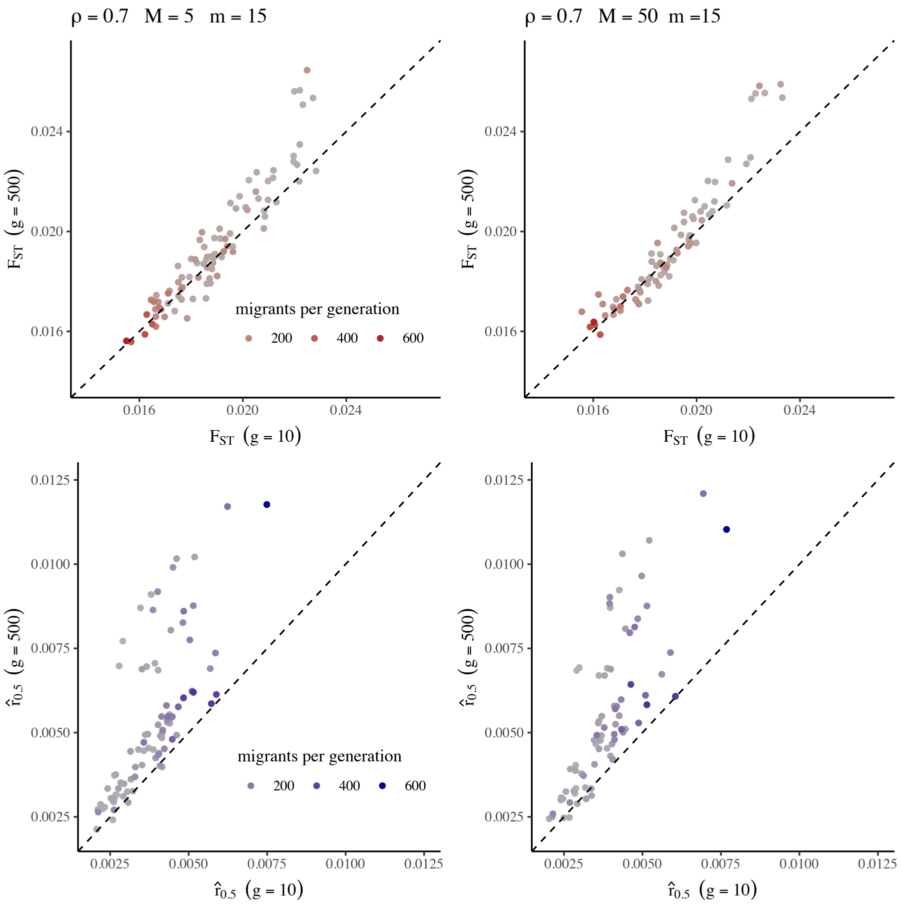

Fig R: Time since ancestral migration events versus $\widehat{F}_{ST}$ or $\widehat{r}_{0.5}$ (Model A). Top panels: Each point compares the $\widehat{F}_{ST}$ value for the same location pair in the simulation using $g = 10$ (x-axis) versus $g = 500$ (y-axis). Bottom panels: Model parameters: M, the multiplier for ancestral migration rates (before g generations in the past), is equal to either 5 (left panels) or 50 (right panels). m, the multiplier for recent migration rates, is equal to 15 and the recombination rate, ϕ, is 0.7 for all simulations shown. Points are colored by the number of migrants per generation in the "recent" migration matrix (from 0 to g generations in the past). The dashed line shows where x-axis and y-axis values are equal.

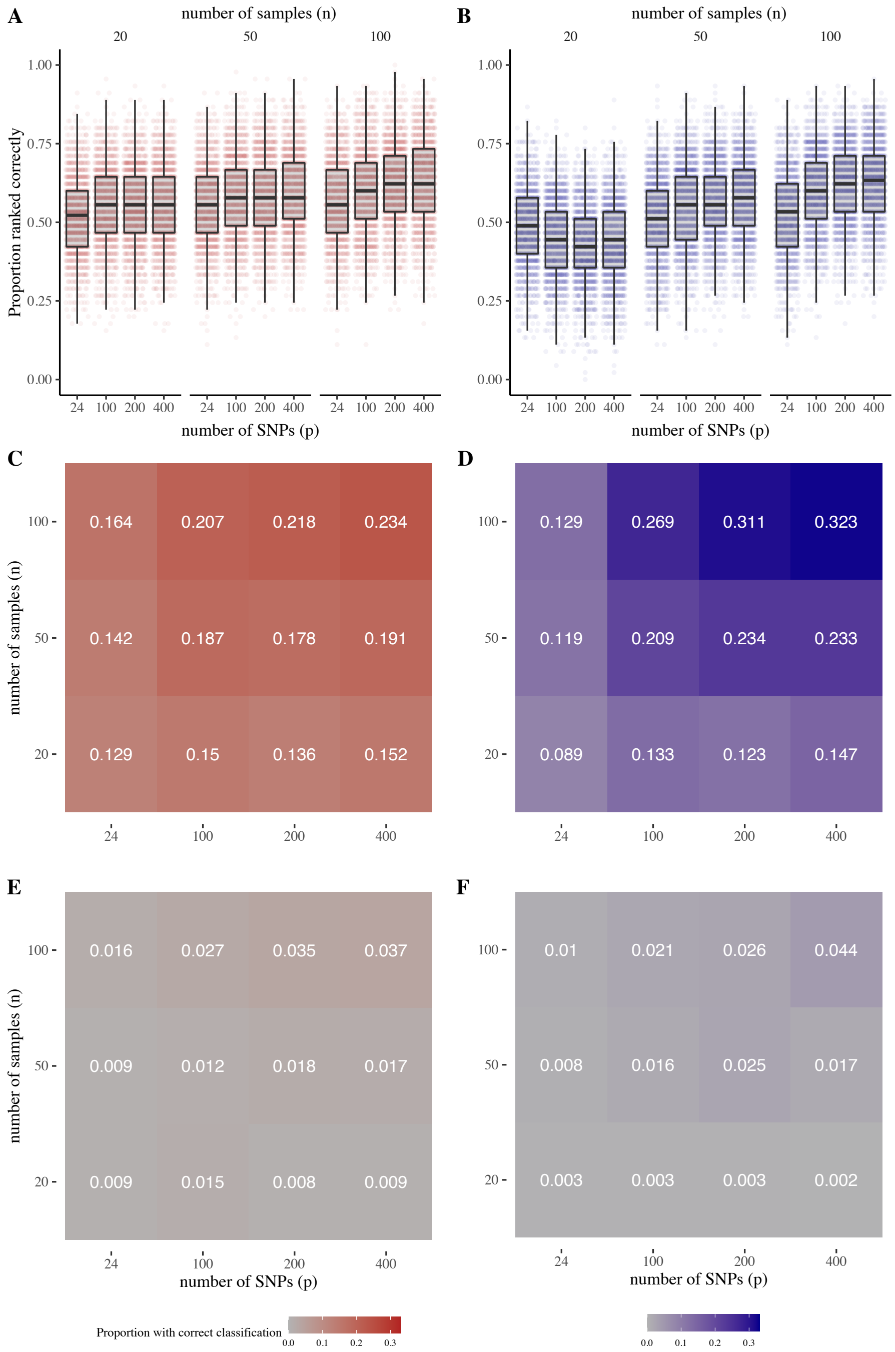

Fig S: Ranking and classification metrics for coalescent-simulated sequence data (local migration, model A). (A) and (B): Proportion of all location-location pairs that are ranked correctly by either F_{ST} or $\widehat{r}_{0.5}$, respectively, when compared to the migration rate specified in the coalescent simulation. Points show these values for 1000 independent simulation replicates over different numbers of individuals (n) and SNPs (p) used to calculate F_{ST} or $\widehat{r}_{0.5}$. (C) and (D): Proportion of all simulation replicates where the location-location pair with the highest migration rate is correctly identified as such by F_{ST} or $\widehat{r}_{0.5}$, respectively. (E) and (F): Proportion of all simulation replicates the location-location pairs with the five highest migration rates are correctly classified as such as such by F_{ST} or $\widehat{r}_{0.5}$.

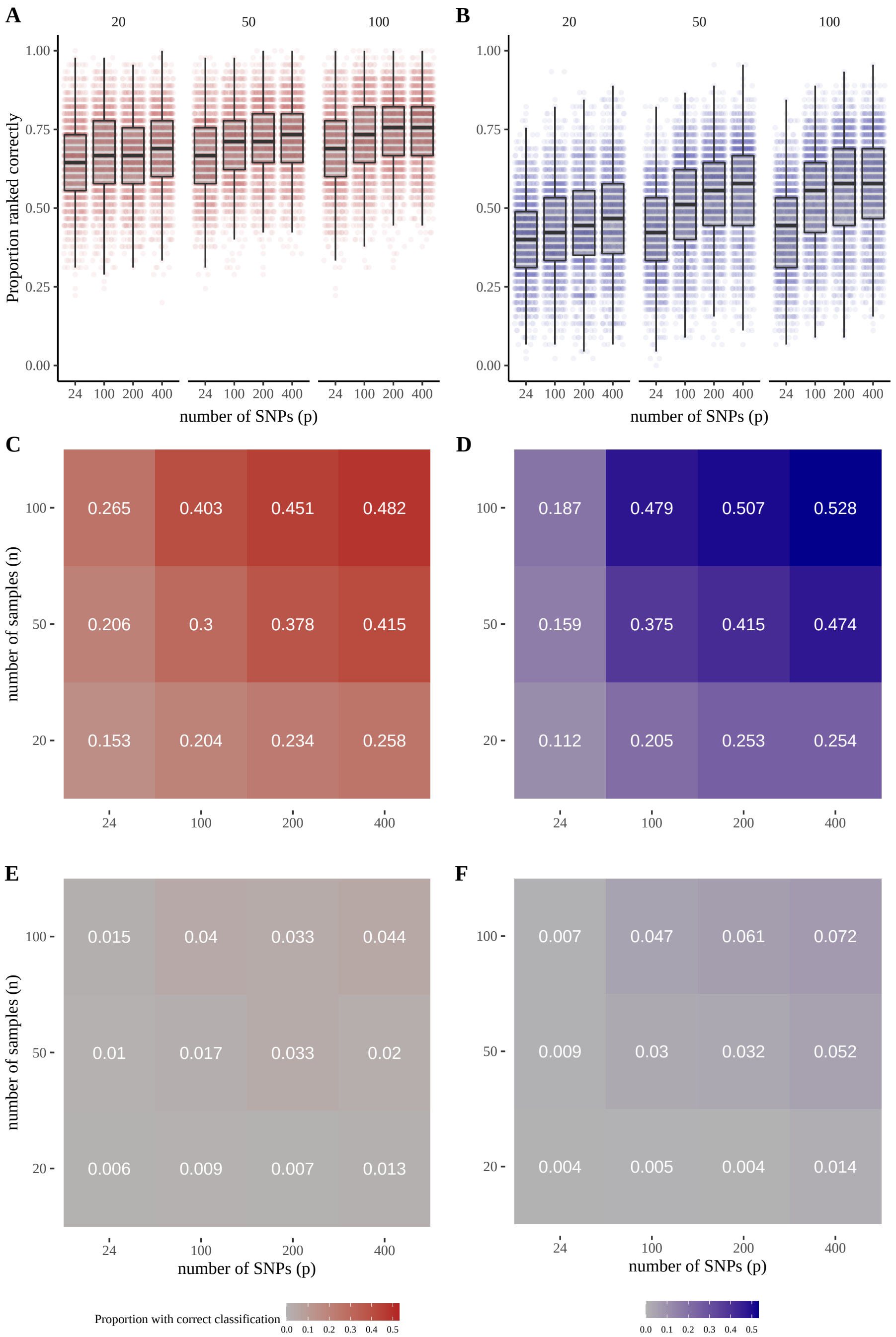

Fig T: Ranking and classification metrics for coalescent-simulated sequence data (subnational migration, model A). (A) and (B): Proportion of all location-location pairs that are ranked correctly by either F_{ST} or $\widehat{r}_{0.5}$, respectively, when compared to the migration rate specified in the coalescent simulation. Points show these values for 1000 independent simulation replicates over different numbers of individuals (n) and SNPs (p) used to calculate F_{ST} or $\widehat{r}_{0.5}$. (C) and (D): Proportion of all simulation replicates where the location-location pair with the highest migration rate is correctly identified as such by F_{ST} or $\widehat{r}_{0.5}$, respectively. (E) and (F): Proportion of all simulation replicates the location-location pairs with the five highest migration rates are correctly classified as such as such by F_{ST} or $\widehat{r}_{0.5}$.

References

[1] Kiang MV, Santillana M, Chen JT, Onnela JP, Krieger N, Engø-Monsen K, et al. Incorporating human mobility data improves forecasts of Dengue fever in Thailand. Scientific Reports. 2021 Jan;11(1). Available from: https://doi.org/10.1038%2Fs41598-020-79438-0.

[2] Brown TS, Engø-Monsen K, Kiang MV, Mahmud AS, Maude RJ, Buckee CO. The impact of mobility network properties on predicted epidemic dynamics in Dhaka and Bangkok. Epidemics. 2021 Jun;35:100441. Available from: https://doi.org/10.1016%2Fj.epidem.2021.100441.

[3] Lloyd CT, Chamberlain H, Kerr D, Yetman G, Pistolesi L, Stevens FR, et al. Global spatio-temporally harmonised datasets for producing high-resolution gridded population distribution datasets. Big Earth Data. 2019 Apr;3(2):108–139.

[4] Joy DA, Feng X, Mu J, Furuya T, Chotivanich K, Krettli AU, et al. Early Origin and Recent Expansion of *Plasmodium falciparum*. Science. 2003;300(5617):318–321. Available from: http://www.jstor.org/stable/3834150.

[5] Anderson TJC, Haubold B, Williams JT, Estrada-Franco§ JG, Richardson L, Mollinedo R, et al. Microsatellite Markers Reveal a Spectrum of Population Structures in the Malaria Parasite *Plasmodium falciparum*. Molecular Biology and Evolution. 2000 Oct;17(10):1467–1482. Available from: https://doi.org/10.1093/oxfordjournals.molbev.a026247.

[6] The Pf3k Project. Pilot data release 5; 2016. www.malariagen.net/data/pf3k-5.
