# Supplementary material for: Distinguishing gene flow between malaria parasite populations
Source: PLoS Genet. 2021 Dec 20;17(12):e1009335. doi: 10.1371/journal.pgen.1009335 (PMC8726502; doi:10.1371/journal.pgen.1009335)
Supplement: Attachment — Submitted filename: plos_genetics_brown_2021.pdf [file pgen.1009335.s002.pdf]

A Review of "Distinguishing gene flow between malaria parasite populations" by Taylor *et al.* for PLOS Genetics

The manuscript sets out to provide an examination of two estimators of population differentiation as proxies for gene flow in the specific context of southeast Asian (SEA) *Plasmodium falciparum* (Pf) populations. The two estimators are the commonly used F_{st}, a standard tool in both population genetics and ecology for estimating the relative heterozygosity of populations and, $\hat{R}_\tau$, an estimate of the proportion of pairs of individuals that exhibit relatedness in excess of τ in a population. The goal of the study is a comparison of two statistics connected to gene flow through an extended worked empirical example and a companion simulation study.

Before getting into the content of my review, let me say that the authors have taken on some substantial ideas in a difficult context and have clearly made some substantial progress in parsing some of them. Understanding gene flow in the context of Pf genetic epidemiology is a hard problem, connecting many types of data, heterogeneously influenced, with models that are not yet well-specified. However, in finding its way to these admirable goals, this manuscript falls short. My critique contains three significant elements: (1) the unncessary framework of attempting to assess which metric is better in some restricted sense; (2) the lack of clear articulation of the contributing factors to gene flow as they relate to these estimators in both the empirical and simulation studies; (3) the lack of clear interpretation of the data set or its contribution to the Pf genetic epidemiology literature. On the one side, it fails as a statistical exploration – is whether $\hat{R}_\tau$ is strictly better than F_{st} in some restricted sense a helpful question if they are both capturing aspects of a complex phenomenon? – and, on the other, it doesn't quite work as an empirical example since, as the authors themselves admit, the data are too heterogeneous in sampling structure to be easily compared in a way that yields insight about the local transmission architecture.

As a point of comparison, many of the authors of this study contributed to the excellent work on the genetic connectivity of Pf on the Columbian cost, also published in PLOS Genetics. There the authors took on an extended empirical case study using very similar tools. However, in that case, the clear goal was the parsing of a particular data set, with a goal of reconstructing the genetic epidemiology. The authors there consider use the tools of this paper together with several other important tools – STRUCTURE, linkage disequilibrium – used in other research to weave together a portrait of the transmission flows. A wide variety of additional tools are cited within the

2017 PLOS Genetics article, where the authors, partially overlapping with those here, articulate the genetic connectivity of a set of Thai/Myanmar data. The artificial restriction to just the two estimators considered in this manuscript strongly limits the richness of its contribution.

The comparison of F_{st} and $\hat{R}_\tau$ as estimators of gene flow

Gene flow is a complex process, and even more so in the context of Pf where within-infection admixture complicates many standard analytic frameworks. While it is surely the case that F_{st} is used as an indicator of gene flow, it is usually understood to be an indirect estimator, since it is effectively measuring the cross-population heterozygosity. While the use of IBD-based metrics relatedness to explore the genetic connectivity between Pf populations has been an exciting, recent site of research, neither does it exactly measure gene flow in a broad sense. In fact, in the paper it's not exactly clear what gene flow means beyond the restricted sense in the simulations and so contextualizing exactly what is being communicated always remains vague within the manuscript. A clear articulation of the contributory factors to gene flow and a delineation of the spatiotemporal scale of its operation in this context is essential for any framework for comparison.

There are many tools to understand the movement of genes across populations that have been deployed in the context of Pf (as mentioned, the wealth of tools cited in the papers above). It is not clearly justified in this manuscript why the restriction to these two estimators is necessary or why only these two estimators are worth comparing. It becomes also clear through the application of the estimators in both the empirical and simulated studies that these are in fact complementary estimators, capturing different aspects of the genetic connectivity that do not need to be understood in opposition. The elaboration of the PRC statistic takes this oppositional comparison to its unnecessary extreme: a statistic that measures the proportion of correct rank between two estimators of a not-clearly-defined quantity. As far as I could find, the mathematical definition of gene flow is made by comparison to the inter-location migration rate in the coalescent simulations (which is fine as far as it goes). But how these particular model parameters connect to the complex structure of Pf gene flow is not clear and not explored. Additionally, the choice of considering scalar $\hat{R}_\tau$ values rather than the more broadly informative distribution of $\hat{r}$ is weakly argued. Figure 1 is one of the most interesting in the paper and details a variety of patterns in the genetic connectivity between the field sites. Generating an artificial threshold statistic for the purpose of comparison with F_{st} seems unnecessary.

The restriction to nearly-equifrequent allele markers in the simulation study makes this comparison particularly awkward. In a previous work, some of the authors have shown that these marker frequency are most efficient for inferring relatedness with HMM-based tools. However, $\hat{F}_{st}$ may not have this property (a discussion of this is not present in the manuscript) so this appears to build up the simulation for the purpose of making one of the estimators perform better by design. One

would tend to think that bias would increase as the number of SNPs increases, possibly explaining a substantial fraction of the simulation results presented.

Another point of clarity for me in the paper overall is the movement between F_{st} and $\hat{R}_\tau$ as the relevant estimates and ΔF_{st} and $\Delta \hat{R}_\tau$ as the controls of their performance. I can see why the performance of both would be controlled by these values but their use in this context and their connection to the primary estimators is poorly explained. Their respective use – rather than finding a unitary control parameter – also prevents any sort of systematic comparison across the two estimators, the exact point the manuscript is motivated to explain.

A final point here is that the study is restricted to monoclonal samples. While no study can do everything, the importance of genetic admixture to Pf gene flow is long-established. Measures of the amount (and structure?) of the polyclonal mixtures in these populations would be another excellent statistical contribution to contextualizing the process of gene flow. If nothing else, some additional attention to the topic in the introduction and discussion would be helpful to the reader to understand the implications that this data restriction may have imposed on the analysis.

The authors mention many of these issues in the Discussion and several straight-forward remedies that can be employed (in fact, seem to have been employed in previous papers). What is unclear is why those steps were not applied here.

A model for gene flow?

There is no clearly articulated model for gene flow in the manuscript, nor is there a discssuion of the many epidemiological factors that can affect it. The closest this work comes to a specification of what gene flow means in a precise sense is the descriptions of the underlying coalescent simulations. That the migration parameter is taken to be equivalent to gene flow is an argument that will need to be articulated more precisely, particularly since the underlying parameters for migration simulation don't appear to be informed by the genetic epidemiology of Pf (it's an arbitrary increasing set of integers). Even granting this connection, it is nowhere considered what this would tell us about the complex gene flow in Pf and how these simulations should be extrapolated to that context.

The absence of an explicit model means that the questions emerging from the estimator comparison become even more obvious. One estimator is then taken to rank better than another if it outperforms the other using the migration parameters as the truth. That this is the correct way to do this is not at all clear, and doesn't take into account other factors (such as population differentiation) that might affect performance in the domain. Even with this ambiguity, the case for the contribution of the $\hat{R}_\tau$ metric is still relatively weak since in many contexts F_{st} seems quite able to provide discrimination. It also makes the transference of the results of the simulation study onto the empirical example difficult.

The data set considered

It might be taken that, simulations and comparison aside, the paper could make a substantial contribution by articulating the transmission structure underlying the samples presented. Certainly, previous studies of genetic epidemiology in the region have been extremely powerful. These studies however have either focused on more localized areas or more regional transmission patterns. This set of samples, combined across several studies and sites, makes for a difficult point of comparison. Since the studies were designed in different ways for different purposes, exactly what these estimates are relating becomes unclear. The authors could shore up this weakness by detailing these various purposes and the biases might bring (if the designs are publicly known) or by taking the sites that have longitudinal surveys and making comparison both within those sites (across years) and then also across sites, providing a natural means for contextualizing gene flow.

Section-specific comments

These are made in addition to the comments above, though overlap somewhat.

1. Introduction

- In general the introduction lays out the questions the study pursues reasonably, but felt a little thin in terms of the background. There have been any number of ways to estimate IBD/IBS in malaria research. Giving the reader a more assured introduction to these previous efforts would help contextualize the work going forward. The papers mentioned above do an excellent job of surveying these works.

- In some sense, the motiat extension of some of the authors' 2018 paper, attempting to fold in questions of migration/gene flow. When people were looking at relatedness (where F_st is a standard measure of population differentiation), the single comparison to the IBD metric seemed reasonable. However, at the next scale up – when we begin to get very complex interactions in terms of geographic/ separation, we also have issues of changing mixture structure, migration patterns involving shifts at the human, vector, and parasite level. A much broader background here would have been helpful.

2. Methods

- Both of these estimates (and their corresponding models) are proxies for gene flow, but neither not direct measures of gene flow. The manuscript somewhat explicitly states this in 2.3. It then changes direction by labelling these metrics (or their pairwise difference) as estimates of gene flow. While imperfect, each contributes some information about gene flow, making a ranking-style statistical comparison not warranted without further motivation. They are not

just two estimators of Wouldn't it perhaps be more integral in this regard to say that the primary goal is to reconstruct gene flow, to which both metrics provide information?

Section 3.1

- Figure 1 gives more evidence that F_{st} and IBD are measuring somewhat different things as they relate to gene flow. The red dots and the blue dots overlap at two pairs, while showing markedly different behavior within the other indicated pairs. This is where the intrinsic better-or-worse comparison is really not serving well: these appear to be complementary approaches.

- Figure S1 B: I'm really not sure what adding the coalescent simulation to the figure is doing, particularly since they are strongly consistent across runs. (This also calls into question exactly how variable the simulations are, and, in turn, how meaningfully they are representing biological context.)

- While the authors note that sampling or population structure could contribute to variable $\hat{r}$, they don't mention polyclonal infections as a factor. While arguably this can be ledgered under population structure, it's contribution to relatedness is a bit different than population structure is usually understood. In specific, an area in which polyclonal infections are rare will necessarily have a high proportion of clonal infections. This has much less to do with broader population structure and much more to do with the host/vector transmission process.

Section 3.2

- Figure 2 again underlines the complementarity of F_{st} and R_{τ} metrics. What I read here is that these two metrics are telling us different things about the overall changes in population structure (which may be sizably affected by gene flow), not that one can be taken to be better than the other. That these metrics are means to capturing overall gene flow but not well-defined estimates of this is further exemplified in Figure S3.

- As a side note on the overall clarity of the paper: the subfigures relating the number of SNPs to the estimates seem superfluous to me, unless the goal is to consider the statistical quality of these estimates, which is not a point of consideration here in my reading. This again goes to the somewhat scattershot structure of the paper – statistical examination, field guide, reconstruction? – that I mention above.

Section 3.3.2

- As an exploration of comparison, this subsection also works to make clear that both estimators are capable of estimating substantial aspects of gene flow that, again, runs counter to the comparative framework the authors have set out.

Section 3.3.3

- This portion of the paper is perhaps the most clear statistical comparison of the two statistics. Again, the authors stratify by the difference in the statistics (which seems again to communicate the importance of those second-order statistics to the broader process of analysis). In my reading of these graphs, counter to what is in the manuscript, I struck by relatively how efficient F_{st} is in capturing differentiation without sophisticated tools like HMMs

- As a point of statistical examination, it's also a bit awkward to compare across the two sets of plots, as they are stratified by delta R and delta F, respectively, and so do not have comparable x-axes. However, if we could make that comparison, it would indicate that the two estimators (again) have complementary properties and that F_{st} frequently is able to discern aspects of the gene flow quite efficiently and often better than $\hat{R}_\tau$. This series of plots also opens again the question of how the instrically summary nature of $\hat{R}_\tau$ interacts with the inference. For instance, if we set τ at 0.95 and 0.8, how do these plots change?
