# Supplementary material for: Distinguishing gene flow between malaria parasite populations
Source: PLoS Genet. 2021 Dec 20;17(12):e1009335. doi: 10.1371/journal.pgen.1009335 (PMC8726502; doi:10.1371/journal.pgen.1009335)
Supplement: Attachment — Submitted filename: BROWN GENE FLOW RESPONSE 091521.pdf [file pgen.1009335.s003.pdf]

Response to reviewers for Brown et al "Distinguishing gene flow between malaria parasite populations"

Reviewer #1: The manuscript attempts to address statistical inference of a single use case of molecular(genomic) epidemiology in malaria control, that is, ranking gene flow between population pairs. Traditionally, this has been done with allele frequency based metrics such as Fst, however here the authors incorporate a more recently established measure of population connectivity, Identity by Descent (IBD) as measured by the parameter R here. Several key findings include that sample size, marker number and population levels and migration influence the ability to rank gene flow.

1. The paper is written well though is a little technical with the mathematics for some general genetics readers, so some effort could be made to simplify some of the descriptions.

We appreciate this general point regarding readability and appropriate level of mathematical detail for the PLOS Genetics audience. We have made multiple changes to address this issue throughout the manuscript and, most importantly, some parts of the manuscript that required more detailed mathematical description have been simplified or removed entirely. Specifically, we have removed the previous approach to evaluating the proportion of location-location pairs that are ranked correctly by either F_{ST} or the IBD-based metric $\hat{r}$ (Section 2.4 in the previous version of the manuscript). The current, revised version of manuscript compares rankings per F_{ST} or $\hat{r}$ versus "ground truth" migration rates specified in the coalescent simulation. We believe that this change simplifies the mathematical content of the paper substantially and overall provides a much more straightforward method for evaluating F_{ST} and $\hat{r}$ as estimates of gene flow between populations. (The latter improvement is discussed further as a response to other reviewer comments below.)

2. One point that stands out for me with IBD statistics is that they rely on high levels of relatedness (near clonality) to measure gene flow. However, these signals will be rapidly lost if a genotype migrated to a high transmission area. Thus, these approaches might be limited when comparing low to low transmission or high to low, but not high to high (as this paper suggests) or low to high transmission. This is something to consider and would be worth commenting on/considering for future analyses.

We agree with the reviewer that differences in levels of transmission between locations is expected to have important impacts on the use of relatedness-based metrics for estimating gene flow. As the reviewer notes, highly-related between-location pairs may have their shared haplotypes broken down quickly if there is high transmission, and thus more frequent recombination, in the location receiving individuals via in-migration. The extent to which differences in transmission in different locations influence estimates of gene flow will also depend on the respective rates of migration between locations, i.e. for two locations *i* and *j*, migration from *i* to *j* and from *j* to *i*.. We have expanded the discussion to include these issues as important topics for further work (lines 365-368), but have not added additional analyses to specifically address these questions. We believe that, given the already expanded scope of the updated manuscript, that dedicated analysis of this question would be best undertaken in future work, unless it is thought essential to understanding the analysis included in the current manuscript.

3. The real data set used was SNPs extracted from genomic data from clinical samples collected in multiple populations of the Greater Mekong Subregion. Here P. falciparum transmission is low and patchy, and finding positive cases is challenging through routine surveys, many thousands of

individuals screened to find only a few samples. Therefore, passive collection of isolates from clinical cases may be the only feasible/affordable means of identifying isolates for genomic analysis. However clinical (febrile) cases, as the authors also point out in the discussion, may present a biased view of transmission dynamics, especially if substantial numbers of asymptomatic infections persist that are not being analysed. There are two issues here, inability to accurately define gene flow due to lack of sufficient samples numbers (also keeping in mind that low density infections are very difficult to genotype currently), and the fact that clinical cases may not present an accurate view of what is actually going on.

We appreciate these insightful comments on additional sources of selection bias (including sampling of clinical vs asymptomatic infections and difficulty sequencing infections with low parasite density). We have added additional text in the discussion highlighting these additional sources of bias (lines 345-347) and underscore these issues as important areas for future research. In particular, we have described under-sampling of subclinical infections as an important issue across a wide range of malaria surveillance applications. Note that we have also more explicitly described how these issues motivate the use of coalescent simulation in our study (given that this approach allows for complete and unbiased sampling of the simulated sequence data). However, we acknowledge that real world surveillance applications have multiple inherent sources of sampling bias and have emphasized this issue in the discussion. We believe our study offers important insights into the use of relatedness- and differentiation-based metrics for estimating gene flow, which can serve as an important step forward for understanding additional issues pertaining to the application of these methods under real-world conditions.

4. The use of the distance by road measures might not be accurate in all settings as in South East Asia, travel by motorbike, boat and foot might be alternative means. Thus a comparison to other distance measures might be warranted. Some of the co-authors have experience in spatial epidemiology so I suspect they have considered this. A comment on why the distance by road method was chosen would be appropriate.

We appreciate this important methodological question and have updated the manuscript to more completely describe our justification for using road distance in this situation. Importantly, at the spatial scale considered in our analysis of the pf3k data (where between-location distances vary from dozens to thousands of kilometers), local methods of transportation (e.g. by foot) are likely less relevant to overall connectivity. Notably, we have overhauled the coalescent simulation used in the manuscript (as described elsewhere in this document and the re-submission cover letter), which now includes the use of mobile phone-associated mobility data to parameterize mobility in the coalescent simulation. In describing the motivation for this analysis, we discuss how geographic distance may be a poor proxy for connectivity in infectious disease applications (lines 57-59) and, in response to this specific reviewer comment, have extended this discussion to include this as a limitation of our analysis of the Pf3k data (lines 354-356).

Reviewer #2: Please find the attached review.

We greatly appreciate the extensive, thoughtful, and detailed comments from Dr. O'Brien. This feedback provided enormously helpful guidance for our revisions, which involved wide-reaching changes to the manuscript and complete re-working of the data analysis included in the paper. Given the extensive nature of these comments, we have here extracted the main feed in the general comments from Dr. O'Brien, which we address in turn below, and then address the section-by-section comments:

General Comments (summarized):

1. "The comparison of F_{ST} and $\hat{R}$ as estimators of gene flow"

In the first section of the general comments, the reviewer offers seven main criticisms: (1) that both F_{ST} and $\hat{R}$ are indirect estimators of gene flow and that "it is not exactly clear what gene flow means [in the paper] beyond the restricted sense in the simulations"; (2) that we have not justified the paper's focus on only F_{ST} and $\hat{r}$ for estimating gene flow; (3) that we pursue unnecessary oppositional comparisons between F_{ST} and $\hat{r}$ rather than examine these as "complementary" estimators of gene flow; (4) that the "proportion ranked correctly" metric is unhelpful (taking "this oppositional comparison to its unnecessary extreme"); (5) that we use a scalar population-level summary of relatedness $\hat{r}$, rather than the entire distribution of $\hat{r}$ values, to examine relatedness between populations; and (6) that we use SNPs with a restricted set of allele frequencies (estimated minor allele frequency > 0.35) and this could result in bias toward improved estimates using $\hat{r}$ rather than F_{ST} ("This appears to build up the simulation for the purpose of making one of the estimators perform better by design."); and (7) that this manuscript does not undertake the issue of polyclonal infections at it relates to estimating gene flow between populations.

We appreciate this detailed and careful analysis of our work and thank reviewer for what standout as incredibly thoughtful and constructive comments.

We view the first limitation noted in (1) as largely unavoidable, i.e. all estimators of gene flow are indirect by nature, but agree with the feedback that what F_{ST} and $\hat{r}$ are estimating needs to be more clearly articulated in the manuscript. We have added text that more explicitly describes what is being estimated in each case: F_{ST} as a proxy for migration that reduces limits the accrual of measurable differentiation between populations and $\hat{r}$ as summary measure of estimated relatedness for between-population individual-individual pairs.

Regarding (2), we focus on F_{ST} given its widespread use in malaria genomic surveillance applications, including a large number important earlier studies and also more recent work. We focus on $\hat{r}$ given the increasing interest and successful application of IBD-based metrics in malaria genomic surveillance applications. Although there are other methods available for both differentiation-based and relatedness-based estimation of gene flow, we would propose that widely evaluating multiple other estimators would potential distract from the focus of the manuscript. We would thus argue that, given the current state of the field and methods currently in use for genomic surveillance applications, evaluating only on F_{ST} and $\hat{r}$ is a justifiable choice. We would argue that this choice focuses our analysis on methods that are most relevant to potential readers from public health and implementation-oriented audiences, where these two estimators are more familiar and widely used. In addition, many of the other available methods for estimating gene flow have thus far had limited practical use in malaria surveillance applications (for example, other differentiation-based metrics like Jost's *D*) and/or are still very early in their development (for example, threshold-free approaches such as Wasserstein distances between parasite populations). We have added text in the discussion on these points (lines 88-90)

We agree with the important feedback provided under point (3) and have used this guidance to substantially revise (indeed, almost entirely re-work) the manuscript. Rather than focus on an oppositional analysis of the "performance" of F_{ST} and $\hat{R}$ with different data sizes or under different epidemiological conditions (as was the focus of the initial manuscript), we have instead refocused the analysis on examining how key features of a population's history, including changes in population size and migration patterns over time, influence the ability to rank estimated gene flow using F_{ST} and $\hat{R}$. This analysis is motivated by the fact that many if not most malaria genomic surveillance efforts are undertaken in contexts where malaria populations are highly dynamic, often in the context of ongoing malaria control interventions. We ground this approach in an updated analysis of the Pf3k data, where we examine F_{ST} and $\hat{R}$ as estimates that can capture gene flow over different time scales. In this updated analysis (Section 3.1 and Figure 1), we show that $\hat{R}$, when used with an appropriately high threshold for identifying highly-related parasite pairs, can be used to capture more recent migration events. F_{ST}, which is informed by genetic variation accrued over a population's entire history, can provide important insights about more ancestral migration events. We extend this analysis using an updated coalescent simulation, informed by mobile phone-associated mobility data from Thailand, that aims to approximate populations with distinctly different migration and population size characteristics in ancestral versus more recent periods in their history (as described in the updated Methods section 2.2). We use human movement, estimated from mobile phone call detail records (CDRs) aggregated over 930 districts in Thailand, to inform this model and examine how mobility over different spatial scales influences F_{ST} and $\hat{R}$. This analysis was undertaken in direct response to Reviewer 2's feedback regarding "a clear articulation of the contributory factors to gene flow and a delineation of the spatiotemporal scale of its operation."

Regarding (4), we have substantially reworked our use of the "proportion ranked correctly" metric in the revised manuscript. In the revised manuscript, we compare rankings per F_{ST} or $\hat{R}$ against the "ground truth" migration rates specified in the coalescent model. We believe this metric captures an important characteristic of F_{ST} and $\hat{R}$ that is relevant for practical use in genomic surveillance applications, i.e. for a given data size (of n individuals and p SNPs), in what proportion of comparisons (between location pairs) does F_{ST} and $\hat{R}$ rank gene flow correctly. We have added additional text in the methods section explaining the justification for using PRC (lines 193-196).

Regarding (5), and the concern that scalar population-level summaries of numerous r values may lose important information about gene flow between populations: we agree that the full distributions of these values are highly informative and, as demonstrated in the pf3k data, offer rich insights into patterns of gene flow between populations. We have included all 36 between-location r value distributions from our analysis of the Pf3k data (nine choose two location pairs, Supplementary Figure S8) and use these results to inform our understanding of gene flow in this data set (Section 3.1). We would note that any comparison between two distributions of r values (for two different location pairs) would likely involve some kind of population-level summary of these distributions, and it is not clear how one would approach the task of ranking or distinguishing levels of gene flow without the use of a population-level summary statistic.

To address point (6), and the concern that restricting our analysis to nearly equifrequent alleles will bias the analysis toward favorable results with $\hat{R}$ rather than F_{ST}, we conducted a focused sensitivity analysis examining this first SNP set (437 SNPs with estimated minor allele frequency >

0.35) and a second SNP set consisting of 5537x` SNPs with estimated minor allele frequency > 0.05. This analysis indicates that both $\hat{R}$ and F_{ST} more reliably capture isolation by distance when using the set of nearly equifrequent alleles (estimated minor AF > 0.35), suggesting that this ascertainment scheme is likely not biased against F_{ST}.

Regarding point (7) and the choice to analyze only monoclonal sequence data: We strongly agree with the reviewer's point on the importance of polyclonal infections for understanding gene flow, particularly in epidemiological contexts where polyclonal infection is common. We have included additional text on this point in the discussion (lines: 347-351). However, we would offer that such issues may be better addressed in future work, focused on how uncertainty and missingness introduced by polyclonal infections influences the ability to estimate gene flow, unless it is thought that this analysis is essential for the current manuscript.

2. "A model for gene flow?"

The second section of general comments provides critical feedback on how the manuscript approaches gene flow from a conceptual standpoint, stating that "There is no clearly articulated model for gene flow in the manuscript, nor is there a discussion of the many epidemiological factors that can affect it." The reviewer also notes some important technical issues with the coalescent model used in the first version of the manuscripts, including the specification of migration rates via sampling from the Dirichlet distribution ("it's an arbitrary increasing set of integers"). We found this feedback particularly instructive for our revisions and used this guidance to rework our approach to the coalescent simulations in the model. Regarding the technical issue of specifying migration rates, the updated manuscript discards the use of the Dirichlet distribution, and instead specifies parasite migration rates using a unique human mobility data set derived from millions of mobile phone call detail records (CDRs) in Thailand. The use of this data, despite requiring the use of several simplifying assumptions about host and parasite movement, has allowed us to more clearly articulate a model for gene flow in the manuscript (in direct response to the reviewer's comments on this point). This model also allows us to evaluate estimated gene flow over different spatial scales, including "local" migration between nearby districts in Thailand and "subnational" migration between more distantly separated districts, which we believe has direct relevance to several important practical questions. We also underscore that the revised modeling approach yields values for F_{ST} and $\hat{R}$ that closely approximate those observed for the Pf3k data.

3. "The data set considered"

In the last section of the general comments, Reviewer 2 highlights several shortcomings of the Pf3k dataset, including the heterogeneous study designs of the studies that contributed isolates to this database. As discussed in our responses elsewhere, and in the updated manuscript (lines 342-345), we also consider this to be an important limitation for the use of the Pf3k dataset here. However, we do not believe this limitation is substantial enough to prevent meaningful interpretation of our results from the Pf3k data, which are largely focused on how differentiation- and relatedness-based estimates can be used to capture gene flow over different periods in a population's history. We have included additional text in the results (lines: 217-239) and discussion (lines: 342-345) on this point, which draws on the within-location r distributions for Pf3k study sites to highlight potential issues with sampling bias in this dataset. The reviewer also

suggests that the paper "the paper could make a substantial contribution by articulating the transmission structure underlying the samples presented." Although we agree that this would be a useful contribution, we believe it would be distinctly outside the stated scope and aims of the paper, which is focused on offering practical guidance for the use of relatedness- and differentiation-based methods for estimating gene flow and the specific analytical task of distinguishing different levels of gene flow (as described in the Introduction).

Section-specific comments (included verbatim)

1. Introduction

• In general the introduction lays out the questions the study pursues reasonably, but felt a little thin in terms of the background. There have been any number of ways to estimate IBD/IBS in malaria research. Giving the reader a more assured introduction to these previous efforts would help contextualize the work going forward. The papers mentioned above do an excellent job of surveying these works.

• In some sense, the motiat extension of some of the authors' 2018 paper, attempting to fold in questions of migration/gene flow. When people were looking at relatedness (where Fst is a standard measure of population differentiation), the single comparison to the IBD metric seemed reasonable. However, at the next scale up – when we begin to get very complex interactions in terms of geographic/ separation, we also have issues of changing mixture structure, migration patterns involving shifts at the human, vector, and parasite level. A much broader background here would have been helpful.

We agree with the Reviewer's comments noting that a broader and deeper introduction to the topics addressed in the manuscript would be helpful. The introduction to the revised manuscript is extensively revised and we believe provides a more comprehensive introduction to the overarching topic, while maintaining a clear focus on the primary aims of the paper: examining how F_{ST} and $\hat{R}$ can be used to distinguish different levels of estimated gene flow between malaria populations, how the ability to distinguish estimated levels of gene flow varies with the amount of data used for estimation, and how different epidemiological conditions may influence the ability to distinguish estimated levels of gene flow.

1. Methods

• Both of these estimates (and their corresponding models) are proxies for gene flow, but neither not [sic] direct measures of gene flow. The manuscript somewhat explicitly states this in 2.3. It then changes direction by labelling these metrics (or their pairwise difference) as estimates of gene flow. While imperfect, each contributes some information about gene flow, making a ranking-style statistical comparison not warranted without further motivation. They are not just two estimators of [sic] Wouldn't it perhaps be more integral in this regard to say that the primary goal is to reconstruct gene flow, to which both metrics provide information?

We appreciate these comments and have included additional text in the manuscript to justify the use of ranking-type comparisons (lines 193-196). We believe that these analyzes provides important information about how reliably values for F_{ST} or $\hat{R}$ can

distinguished from one another at different data sizes and under different epidemiological conditions. From a practical standpoint, focused on how results from genomic surveillance studies can guide malaria control interventions and public health decision making, we would propose that reliably distinguishing and ranking observed values F_{ST} or $\hat{R}$ is a centrally important task. Consider these questions from the standpoint of a malaria control program manage reviewing results from a genomic surveillance study: Given the amount of data used in the study (number of loci genotyped and number of individuals sampled), how confident should one be that different F_{ST} or $\hat{R}$ values are truly indicative of different levels of gene flow between location pairs? We have included additional information on this practical, field-level motivation for focusing on ranking and distinguishing F_{ST} or $\hat{R}$ values. As noted before, we have undertaken a major effort to move away from direct comparisons between F_{ST} and $\hat{R}$, with more focus on the complementary information offered by the two approaches. However, with the aforementioned practical motivation, these comparisons are to a certain extent unavoidable.

Section 3.1

• Figure 1 gives more evidence that Fst and IBD are measuring somewhat different things as they relate to gene flow. The red dots and the blue dots overlap at two pairs, while showing markedly different behavior within the other indicated pairs. This is where the intrinsic better-or-worse comparison is really not serving well: these appear to be complementary approaches.

We agree with this constructive feedback and have expanded substantially on these points in the revised manuscript, including additional analysis of within- and between-location *r* distributions (Supplementary Figures S8 and S9) and analysis of pairwise shared IBD length (Supplementary Figure S7). We use findings from this first section of results in the new manuscript (Section 3.1) to motivate and inform our updated modeling approach, which seeks to examine F_{ST} or $\hat{R}$ in a coalescent model that more accurately approximates the dynamic nature of real-life malaria populations, and the ancestral and more recent events influencing genetic signatures of gene flow.

• Figure S1 B: I'm really not sure what adding the coalescent simulation to the figure is doing, particularly since they are strongly consistent across runs. (This also calls into question exactly how variable the simulations are, and, in turn, how meaningfully they are representing biological context.)

This figure is no longer included in the manuscript. Given the extensively re-worked (i.e. entirely new) modeling approach, this figure is no longer relevant.

• While the authors note that sampling or population structure could contribute to variable r̂, they don't mention polyclonal infections as a factor. While arguably this can be ledgered under population structure, it's contribution to relatedness is a bit different than population structure is usually understood. In specific, an area in which polyclonal infections are rare will necessarily have a high proportion of clonal infections. This has much less to do with broader population structure and much more to do with the host/vector transmission process.

As noted above, we have added additional text regarding the importance of polyclonal infections (and how the exclusion of polyclonal infections may limit the generalizability of our findings) in the discussion (lines 347-351)

Section 3.2

• Figure 2 again underlines the complementarity of Fst and Rτ metrics. What I read here is that these two metrics are telling us different things about the overall changes in population structure (which may be sizably affected by gene flow), not that one can be taken to be better than the other. That these metrics are means to capturing overall gene flow but not well-defined estimates of this is further exemplified in Figure S3.

We agree with these comments and the overall point that F_{ST} or $\hat{R}$ values offer complementary information on overall patterns of gene flow between populations. These comments motivated a revised examination of these estimators throughout the revised manuscript, focused on how F_{ST} and $\hat{R}$ can be used to capture genetic signatures of migration events occurring at over both ancestral and more recent time scales (as discussed elsewhere in these responses)

• As a side note on the overall clarity of the paper: the subfigures relating the number of SNPs to the estimates seem superfluous to me, unless the goal is to consider the statistical quality of these estimates, which is not a point of consideration here in my reading. This again goes to the somewhat scattershot structure of the paper – statistical examination, field guide, reconstruction? – that I mention above.

We appreciate this candid feedback and have taken extensive efforts to clarify the analytical goals of the paper and the motivation for the kinds of analysis (i.e. ranking and distinguishing gene flow). To reiterate, this paper aims to provide practical guidance for the design and interpretation of malaria genomic surveillance studies, with the specific goal of supporting the use of these studies for practical decision-making in malaria control programs. From this standpoint, questions about data size (including numbers of SNPs used and number of individuals sampled) are centrally important and thus receive significant attention in the manuscript.

Section 3.3.2

• As an exploration of comparison, this subsection also works to make clear that both estimators are capable of estimating substantial aspects of gene flow that, again, runs counter to the comparative framework the authors have set out.

Please see our responses for Sections 3.1 and 3.2 and our responses to these points in the general comments.

Section 3.3.3

• This portion of the paper is perhaps the most clear statistical comparison of the two statistics. Again, the authors stratify by the difference in the statistics (which seems again to communicate the importance of those second-order statistics to the broader process of analysis). In my reading of these graphs, counter to what is in the manuscript, I struck by

relatively how efficient Fst is in capturing differentiation without sophisticated tools like HMMs

The revised manuscript uses an extensively revised coalescent simulation framework and no longer includes this model used for generating the results in Section 3.3.3. However, results from similar analyses, examining data size requirements but using the updated modeling framework, are detailed in Section 3.3 of the revised manuscript.

• As a point of statistical examination, it's also a bit awkward to compare across the two sets of plots, as they are stratified by delta R and delta F, respectively, and so do not have comparable x-axes. However, if we could make that comparison, it would indicate that the two estimators (again) have complementary properties and that Fst frequently is able to discern aspects of the gene flow quite efficiently and often better than Rˆ τ . This series of plots also opens again the question of how the instrically summary nature of Rˆ τ interacts with the inference. For instance, if we set τ at 0.95 and 0.8, how do these plots change?

The updated manuscript no longer uses ΔF_{ST} or $\Delta\hat{R}$ to stratify results in the figures, but instead uses known migration rates (pre-specified in the coalescent simulation) throughout the analysis and presentation of results. This provides a much clearer unitary control parameter for the comparison of results obtained using F_{ST} versus $\hat{R}$.

Reviewer #3: This is a well written manuscript describing approaches for measuring geneflow between populations, connectivity. It addresses an important question relevant to malaria elimination and the translation of genomic surveillance approaches for this deadly disease. It contrasted measures of geneflow based on allele frequencies (FST) and ancestry (measured by an IBD index, R). They employed real and simulated data, the latter controlling for migration rates and parameters that remain vastly unknown in natural parasite populations. Despite the importance of the findings, the manuscript is limited by a by the following:

1. The authors deliberately used data from the Greater Mekong region and only monoclonal isolates. This is convenient but far from the reality across malaria endemic regions. With the bulk of malaria transmission in Africa, there should be strong motivation to employ such approaches in this population especially for populations with reduced transmission, where monoclonality is high and elimination plans are being developed. Overall, how this will apply to more complex populations need further discussion.

We strongly agree with the reviewer on this point and appreciate the underlying motivation, i.e. how do we apply malaria genomic surveillance tools in contexts where malaria is most highly endemic, where malaria populations are inherently more complex and potentially more difficult to study with existing genomic tools? We agree that polyclonal infections are a critically important issue for genomic surveillance applications in these contexts and have included additional text on this issue in the discussion. We would offer that such issues may be better addressed in future work, focused on how uncertainty and missingness introduced by polyclonal infections influences the ability to estimate gene flow, and whether approaches for identifying individual strains from polyclonal sequence data can improve gene flow estimates in these contexts.

2. As recognised by the authors, the bias in sampling is a major issue for connectivity studies based on genetic data. The authors could discuss the best sampling strategy for capturing connectivity at high confidence

We thank the reviewer for this comment and the opportunity to provide additional guidance in the manuscript. We have added text to the discussion in this point, which describes which sampling modalities are expected avoid these potential sources of bias, specifically randomized sampling approaches that capture both clinical and subclinical infections.

3. The simulations allow for only 100 individuals per location without any justification for this number.

We appreciate the feedback on this point. We have added text to the methods section explaining this choice, which is largely used as a simplifying assumption for an analysis that looks across numerous combinations of multiple parameters. To clarify, the 100 individuals sampled in this case refer to the number of individual sequences sampled per location (i.e. subpopulation) in the coalescent simulation. This group of 100 individuals provides the sequence data from which we randomly sselect individuals of sample size n for our evaluation of data size requirements. We note also that, for the coalescent simulation, we use balanced sampling (n individuals from each location) and do not explore F_{ST} and $\hat{r}$ when sampling is unbalanced (i.e. for locations i and j, $n_i \neq n_j$). This is a simplifying assumption intended to focus the analysis, but does suggest an important area for additional work. We would offer that this analysis is better left for future work, unless it is thought to be needed for understanding the analysis included in the current manuscript.

4. The filtered data retained 418 SNPs already biased for high minor allele frequencies. This will affect measurement of FST as low frequency variants are eliminated. Again, the choice on 0.35 MAF is based on a previous reference.

The updated manuscript includes a new sensitivity analysis examining the influence of allele frequency on gene flow estimates and evidence of spatial population structure. This analysis compares the previously used SNP ascertainment scheme (n=437 SNPs with estimated minor allele frequencies > 0.35) versus a less stringent SNP ascertainment scheme that permits this inclusion of more rare alleles (5537 SNPs with estimated minor allele frequencies > 0.05). This analysis suggests that the ability to resolve different levels of gene flow, using either F_{ST} or $\hat{R}$, is decreased when using the second ascertainment scheme. For the remaining analyses in the paper, we use the more stringent threshold, given that (1) this improves the ability to resolve different levels of gene flow for both F_{ST} or $\hat{r}$ estimates and (2) is consistent with ascertainment schemes used commonly in genomic surveillance applications.

5. The index for pairwise relatedness is considered a summary for the genomes of the pairs. This will be true if the 418 SNPs are well distributed across all chromosomes. Except I missed this, data on how the retained loci span across the genome should be helpful.

We have added information on the distribution of SNPs from this ascertainment scheme in the supplementary material.

6. Authors excluded sequences carrying the KEL1 haplotype. Connectivity studies as indicated in

their introduction will help also to determine how antimalarial resistance flows between populations. Instead of excluding the isolates with KEL1 haplotypes, removal of SNPs around drug resistant gene sweeps could allow for tracking the flow of drug resistant isolates.

We agree with this insight and with the broader observation that including drug resistance-associated haplotypes, or potentially restricting gene flow estimation to only markers within these haplotypes, could provide useful information about connectivity and geographic dispersal of antimalarial resistance-associated genes. The revised manuscript includes a note on this point in the discussion (lines 351-354) We excluded parasites carrying KEL1 haplotypes associated with resistance, and excluded samples from all years when these haploytypes were widespread across the pf3k study area, in order to focus on underlying patterns of gene flow, outside of these mediated by selection for artemisinin resistance.

7. The Ne estimates used were from an African population in Senegal, obtained after years of interventions. Not sure they apply to SEA, and why this not determined. Determining Ne accurately is an issue as with other popgen parameters.

The revised manuscript includes an updated coalescent modeling framework that we believe is better suited for studying gene flow estimates in real-world populations and for more comprehensively understanding how estimates based on relatedness and differentiation are influenced by changes in migration rates and population sizes over time. This is motivated by the fact, as highlighted by the reviewer, that malaria population sizes in most contexts are dynamic over time, particularly in populations that are subjected to effective malaria control interventions (for example, those in Senegal or the Thai-Myanmar border). We use two models in this framework: one in which population size decreases over time, from an initial or ancestral population size of N_i to current or final population size N_f, and the matrix of migration rates between locations is constant over time ("Model A"); and a second model in which the migration matrix changes from a set of ancestral migration rates to a (different) set of current migration rates at time = g generations in the past, with the effective population size constant over time ("Model B"). We used a final effective population sizes of 100 and 500 in models A and B and, in model B, use ancestral population sizes of 1000 or 5000 sequences. Figures S9-S11 compare the distribution of model-generated F_{ST} or $\hat{r}$ against values observed in the Pf3k data. We believe that these parameter choices provide adequate approximations for population sizes in real-world malaria populations. Moreover, we note that there is considerable uncertainty in estimated effective sizes of real-world malaria populations, such that attempting to exactly replicate published estimates for N_e in our model could result in misspecification or, at least, over-reliance on a parameter for which there is no consensus "ground truth" value (and that varies widely across epidemiological settings). Inclusion of the aforementioned sensitivity analysis allows for some insights into how population size is expected to change under different population size scenarios, even if the true values of these parameters are not well known.

8. Phi was not defined and the value of 0.044 was used to scale the recombination rate. Only specialised readers will get this for an approach intended for future wider translation. How was this derived?

We appreciate this comment and appreciate the careful review of our modeling procedures. We note that recombination rates in naturally-occurring populations are difficult to estimate, subject to uncertainty and controversy, and also mediated by facultative self-fertilization (which is driven by population size and diversity). In our updated modeling framework, rather than attempt to stringently

specify recombination rates (and risk mis-specification by over-reliance on potentially inaccurate estimates for this parameter) we chose to evaluate a range of plausible recombination rates and describe the influence of this parameter on resulting estimates of gene flow (similar to our approach to specifying population size, as described above). We provide a sensitivity analysis across different recombination rates in Supplementary Figures S13 and S14.

9. What is the added value of PRC, given R seems a robust measure of connectivity?

As noted in response to Comment #1 from Reviewer #1, we have re-worked the use of the PRC ("proportion ranked correctly") metric in the manuscript. In the revised manuscript, we compare rankings per F_{ST} or $\hat{R}$ against the "ground truth" migration rates specified in the coalescent model. We believe this metric captures an important characteristic of F_{ST} and $\hat{R}$ that is relevant for practical use in genomic surveillance applications, i.e. for a given data size (of n individuals and p SNPs), in what proportion of comparisons (between location pairs) does F_{ST} and $\hat{R}$ rank gene flow correctly. We have added additional text in the methods section explaining the justification for using PRC (lines 193-196).

10. FST was not displayed on figure 1 to help readers

The figures in the updated manuscript have changed substantially and we have carefully reviewed all the new figures for formatting or display issues.

11. R is highly skewed. Low FST but high R will result from the presence of highly related pairs, which can be due to a common source outbreak rather than connectivity.

We appreciate this insightful observation. We agree that a common source outbreak in one location, with subsequent migration of these highly clonal parasites into other locations would likely result in larger numbers of highly-related individual-individual pairs shared between the outbreak source location and locations linked to it via migration. This could skew metrics based on relatedness (and also divergence) toward higher estimated levels of gene flow (when compared to similar levels of parasite migration from a more diverse source population). This question is related to those raised in Comment #2 from Reviewer #1, which queries how differences in population size and diversity between locations influence estimated levels of gene flow between locations. We have added text on these two questions to the Discussion section (lines 365-368). As noted above, we would propose that additional analysis on these questions is best left for future work, unless it is thought that examining them is essential to the current manuscript – in which case we would gladly take the opportunity to expand on our work.
