# Supplementary material for: Distinguishing gene flow between malaria parasite populations
Source: PLoS Genet. 2021 Dec 20;17(12):e1009335. doi: 10.1371/journal.pgen.1009335 (PMC8726502; doi:10.1371/journal.pgen.1009335)
Supplement: Attachment — Submitted filename: plos_genetics_brown_first_revision_2021.pdf [file pgen.1009335.s004.pdf]

Review of "Distinguishing gene flow between malaria parasite populations" by Brown *et al.* for PLOS Genetics

Just for context, the revised manuscript examines two estimators of population differentiation as tools for estimating gene flow, using data from southeast Asian (SEA) *Plasmodium falciparum* (Pf) populations as well as extensive coalescent simulations. The two estimators are the widely-used F_{st}, a workhorse population genetics and ecology for estimating the genetic divergence between populations, and $\hat{r}_\tau$, an estimate of the proportion of individual pairs showing a relatedness in excess of τ, as estimated by an HMM model. The study seeks to examine the complementary performance of these two statistics as a way to contextualize gene flow through the empirical and numerical examples.

First off, I'm deeply appreciative of the attention the authors have shown to my comments. I apologize if my review was unduly harsh: while I think this work contributes to the statistical genetic literature around Pf genetic epidemiology, the direct comparison between F_{st} and r as metrics where one could be termed 'better' did not sit well with me. Reading through the paper now, I'm quite impressed with the even-handed way in which the authors have situated and compared the two estimators and given useful context to their use in a difficult but applicable example (SEA). The thorough simulation study underscores many of their points of use – number of SNPs to be used, the complementary aspects of the two estimators – and successfully builds out a paper that I find to be much more helpful in guiding researchers in the use of these tools.

I find the revised manuscript to be quite well-written and easy to parse. The comments that address the reviewers points of concern were thoughtful and I thought ably handled the issues raised, including my own. The authors have struck a good balance between the genetic presentation and the statistical concerns in a way that will be of use to genetic researchers and of interest to more statistically minded readers. The multipaneled figures (e.g. FIgure 1) showing how F_{st} compares against $\hat{r}$ for different τ values in the context of SEA were excellent and provided a lot of information about the relative connectivity of these populations.

Points for consideration

Below I note a few small concerns to aid the authors in perhaps clarifying some points that I found difficult to follow in the manuscript. However, each of these is minor and should not in any way preclude publication.

- Since the comparison between relatedness and differentiation is one that the authors rely on in the manuscript repeatedly, it would be helpful to have this distinction laid out more clearly early in the manuscript (which is not to say that it's poorly done as written, just that it's an important point that could be articulated). Perhaps it might be helpful to the reader to add a sentence noting that the comparison between relatedness-based and differentiation-based estimates (in the context of Pf) is the core theme of this paper?
- In the third paragraph, the authors note that ranking regions in terms of genetic connectivity is an important use case. Some citations would add heft to this assertion.
- In FIgure 1, each of the panels contains a color-graded index (for instance, F_{st} goes from 0.08 to 0.0128). Are these necessary? It would seem that this is the same information that is being presented is already in the plot itself. The color gradient suggests additional information that is (if I'm reading things correctly) presenting the same information twice?
- Are the values of the Mantel test necessary in Figure 1 (and others)? Could these not be removed to the manuscript? They are a little confusing in the figure (although clearly noted and described) and not necessarily adding to the story therein.
- This is maybe more of wish than a suggestion but I would have loved to have seen the equivalent of Figure 1 (b) and (c) with $\hat{r}_\tau$ for τ varying over more values than just 0.1 and 0.5. Perhaps this would make for an interesting Supplementary Figure? For me, such a figure would be a very interesting exploration of how relatedness connects to spatial connectivity that the current Figure 1 details nicely, if sparsely.
- A different but related wish would be to see how F_{st} plots against $\hat{r}_\tau$. This would allow the reader to see how these metrics compare in the SEA context and then see for themselves how F_{st} provides long-range information while $\hat{r}_\tau$ provides local, granular information.
- I thought the graphical description of the models (Supplementary Figure 3) was very helpful in understanding the design of the simulations and thought it could be profitably 'promoted' to a full figure if the authors thought that would be helpful. Similarly, I thought that FIgure 4 could be relegated to the supplementary information.
